# Effect Modification by Ambient Temperature on the Association of Ambient Ozone Exposure with Diet-Controlled and Insulin-Treated Gestational Diabetes Mellitus

**DOI:** 10.3390/toxics14070622

**Published:** 2026-07-16

**Authors:** Yuanyuan Yu, Sujuan Hou, Yifei Li, Yajuan Wang, Qisijing Liu, Qirong Zhang, Shijun Ni, Chen Li, Liqiong Guo, Cha Han

**Affiliations:** 1 Department of Obstetrics and Gynecology, Tianjin Medical University General Hospital, Tianjin 300072, China; 2 School of Disaster and Emergency Medicine, Tianjin University, Tianjin 300072, China; 3 Wenzhou Safety (Emergency) Institute, Tianjin University, Wenzhou 325000, China; 4 Tianjin Key Laboratory of Disaster Medicine Technology, Tianjin 300072, China; 5 Tianjin Key Laboratory of Female Reproductive Health, Tianjin Medical University General Hospital, Tianjin 300072, China; 6 Research Institute of Public Health, School of Medicine, Nankai University, Tianjin 300350, China; 7 Department of Occupational and Environmental Health, School of Public Health, Tianjin Medical University, Tianjin 300070, China; 8 Tianjin Key Laboratory of Environment, Nutrition and Public Health, School of Public Health, Tianjin Medical University, Tianjin 301700, China

**Keywords:** ambient ozone, ambient temperature, gestational diabetes mellitus, effect modification

## Abstract

**Background**: Previous studies have explored the association between ambient ozone (O_3_) exposure and gestational diabetes mellitus (GDM). However, little is known about the association of O_3_ with different GDM clinical classifications—diet-controlled GDM (GDMA1) and insulin-treated GDM (GDMA2), and whether temperature modifies the associations. **Methods**: We conducted a retrospective cohort study including 11,491 pregnant women between 2017 and 2023 in Tianjin, China. O_3_ exposure levels for each participant were assessed using the Tracking Air Pollution in China dataset. Logistic regression models were employed to analyze the associations of trimester-specific O_3_ exposure with GDMA1 and GDMA2. Distributed lag nonlinear model (DLNM) was used to identify potential critical gestational weeks of O_3_ exposure. Ambient temperature was categorized using the 10th and 90th percentiles to evaluate the effect modification of extreme temperatures on these associations. **Results**: No statistically significant association was observed in the fully adjusted trimester-specific models. In the weekly-specific DLNM analyses, we observed 5–12 weeks before pregnancy and 26–28 gestational weeks as sensitive windows where O_3_ exposure was weakly associated with elevated GDMA1 risk, and 11–25 gestational weeks for GDMA2. Each 10 μg/m^3^ increase in O_3_ during pregnancy was associated with small increases in the odds of GDMA1 (28 weeks: OR = 1.019, 95% CI: 1.002, 1.035) and GDMA2 (25 weeks: OR = 1.018, 95% CI: 1.002, 1.035). High temperatures appeared to strengthen the observed association between preconception O_3_ exposure and GDMA1. Additionally, O_3_ exposure during pregnancy may increase the risk of GDMA1 among women aged <35 years, preconception obesity, gestational hypertension, and family history of diabetes. **Conclusions**: This study suggests possible weak weekly-specific associations between O_3_ exposure and GDM classifications and indicates that ambient high temperature may modify the O_3_-GDM association. These findings should be interpreted cautiously and require confirmation in future studies.

## 1. Introduction

Gestational diabetes mellitus (GDM) is defined as the first onset of glucose intolerance during pregnancy. As a common pregnancy complication, it has been recognized as a major public health concern worldwide [1]. The global prevalence of GDM is estimated at 14.0% according to the International Association of the Diabetes and Pregnancy Study Groups (IADPSG) criteria, with a notably rising trend observed in low-income countries, particularly in the Middle East and Southeast Asia [2]. Clinically, most GDM patients can achieve blood glucose control through a balanced diet and are classified as diet-controlled GDM (GDMA1). However, pharmacologic intervention (e.g., insulin) is required for patients whose blood glucose fails to meet targets, and these patients are termed insulin-treated GDM (GDMA2) [3]. Regardless of glycemic control, GDM elevates the risk of adverse health outcomes for both the mother and infant [4,5]. Short-term complications include adverse perinatal outcomes such as macrosomia, preeclampsia, and birth trauma [6]. Long-term risks are characterized by an increased susceptibility to metabolic disorders and cardiovascular diseases in both mothers and children [7,8]. Notably, GDMA2 is associated with a higher risk of adverse perinatal outcomes compared to GDMA1 [9]. Given the substantial health and economic burden imposed by GDM, identifying modifiable risk factors and elucidating their differential effects on GDMA1 and GDMA2 are crucial for developing targeted prevention strategies.

Beyond well-established GDM risk factors (e.g., advanced maternal age, preconception obesity, family history of diabetes), ambient air pollution—ozone (O_3_), especially, has been identified as a potential environmental risk factor for GDM [1,10]. The 2019 Global Burden of Disease (GBD) Study reported that O_3_ exposure contributed to approximately 365,000 premature deaths globally [11]. Additionally, ambient O_3_ concentrations have continued to rise in recent years, a trend particularly pronounced in China [11,12]. Although several studies have explored the association between O_3_ exposure and GDM, the findings remain inconsistent [10,13,14,15,16,17,18,19]. Some cohort studies have reported that preconception or prenatal O_3_ exposure is associated with an increased risk of GDM [10,17,18,19], potentially mediated through β-cell dysfunction, systemic inflammation, oxidative stress, neurohormonal dysregulation, and insulin resistance [20]. However, other studies have found no significant association or even suggested a protective effect [15,16]. These discrepancies may be attributed to variations in exposure assessment methods, definitions of exposure windows, and differences in geographical and population characteristics. Moreover, most previous studies predominantly focus on long-term exposure estimates, lacking refined temporal analyses needed to identify high-risk sensitive windows for the O_3_-GDM association. Meanwhile, existing studies have generally treated GDM as a homogeneous outcome without distinguishing between its clinical classification, thereby limiting insights into whether O_3_ exposure affects GDM severity differentially.

O_3_ is a typical photochemical oxidant formed primarily through reactions between nitrogen oxides and volatile organic compounds under sunlight and high temperature conditions [21]. Intense solar radiation and high temperatures can further increase O_3_ concentrations during extreme heat events. Compared with the independent effects of individual factors, the interaction between high temperature and high O_3_ concentrations may jointly affect human health [22]. Previous studies have indicated that prenatal high temperature exposure is associated with an elevated risk of GDM [23,24,25]. However, temperature was mostly adjusted as a confounder in the association between O_3_ exposure and GDM [13,17,19]. Only limited studies have explored the effect modification of temperature on the association between O_3_ exposure and health outcomes, including adverse pregnancy outcomes [26,27], and cardiovascular hospitalizations and mortality [28,29]. Therefore, clarifying whether temperature modifies the association between O_3_ exposure and GDM may help improve prevention and control strategies for pregnancy health risks.

To address these research gaps, this study investigated the associations between O_3_ exposure and different GDM classifications (GDMA1 and GDMA2) among 11,491 pregnant women from a birth cohort in Tianjin, China. Additionally, we evaluated the effect modification of ambient temperature on these associations and further identified sensitive windows and vulnerable populations.

## 2. Materials and Methods

### 2.1. Study Design and Population

This study recruited participants at Tianjin Medical University General Hospital from 2017 to 2023. All participants were permanent residents of the 16 districts in Tianjin and were followed up until delivery. Professional medical staff collected information including demographic characteristics, lifestyle, medication use during pregnancy, and clinical examination data through standardized assessments. Exclusion criteria included: maternal age < 18 years, missing address information or non-permanent residence in Tianjin, gestational age outside the 24–42 week range, non-singleton live births, a history of chronic hypertension, heart disease, diabetes, hepatic/renal disease, epilepsy, or infectious diseases [30], and those who could not be diagnosed with any classification of GDM. Finally, a total of 11,491 pregnant women were included in the analysis (Figure S1). This study was approved by the Ethics Committee of Tianjin Medical University General Hospital (Approval No: IRB2024-YX-101-01). All participants signed a written informed consent form after fully understanding the purpose and procedures of the study.

### 2.2. Outcome Assessment

All pregnant women underwent a 75 g oral glucose tolerance test (OGTT) between 24 and 28 weeks of gestation. GDM was diagnosed according to the IADPSG criteria [31]. Diagnosis required meeting at least one of the following conditions: (1) fasting blood glucose ≥ 5.1 mmol/L; (2) 1 h blood glucose ≥ 10.0 mmol/L after oral administration of 75 g of glucose; (3) 2 h blood glucose ≥ 8.5 mmol/L after oral administration of 75 g of glucose. According to the method of blood glucose control, GDM was further classified into GDMA1 and GDMA2 [3]. Specifically, GDMA1 was defined as GDM adequately controlled through diet alone (without medication), while GDMA2 referred to GDM requiring insulin therapy during pregnancy to maintain blood glucose control [32,33].

### 2.3. Exposure Assessment

Daily maximum 8 h average (MDA8) O_3_ concentration data for the study period (2017–2023) were obtained from the Tracking Air Pollution (TAP) dataset in China (http://tapdata.org.cn/). This dataset provides near-real-time gridded data with a spatial resolution of 10 km × 10 km, which is generated by fusing ground observations, Community Multiscale Air Quality (CMAQ) simulations, Ozone Monitoring Instrument (OMI) satellite O_3_ profiles, Modern-Era Retrospective analysis for Research and Applications, Version 2 (MERRA-2) meteorological parameters, Moderate Resolution Imaging Spectroradiometer (MODIS) Normalized Difference Vegetation Index (NDVI), and National Centers for Environmental Information (NCEI) annual night light data [34,35]. A 5-fold cross-validation was conducted to evaluate the performance of O_3_ concentration calculation, and the results showed that the cross-validation coefficient of determination (R^2^) was 0.84 [34], indicating high prediction accuracy. Daily average temperature and dew point data were acquired from Tianjin monitoring stations through the China Meteorological Data Service Center (http://data.cma.cn/). Details of the exposure assessment for such meteorological data have been described in previous studies [36].

**Figure 1:**
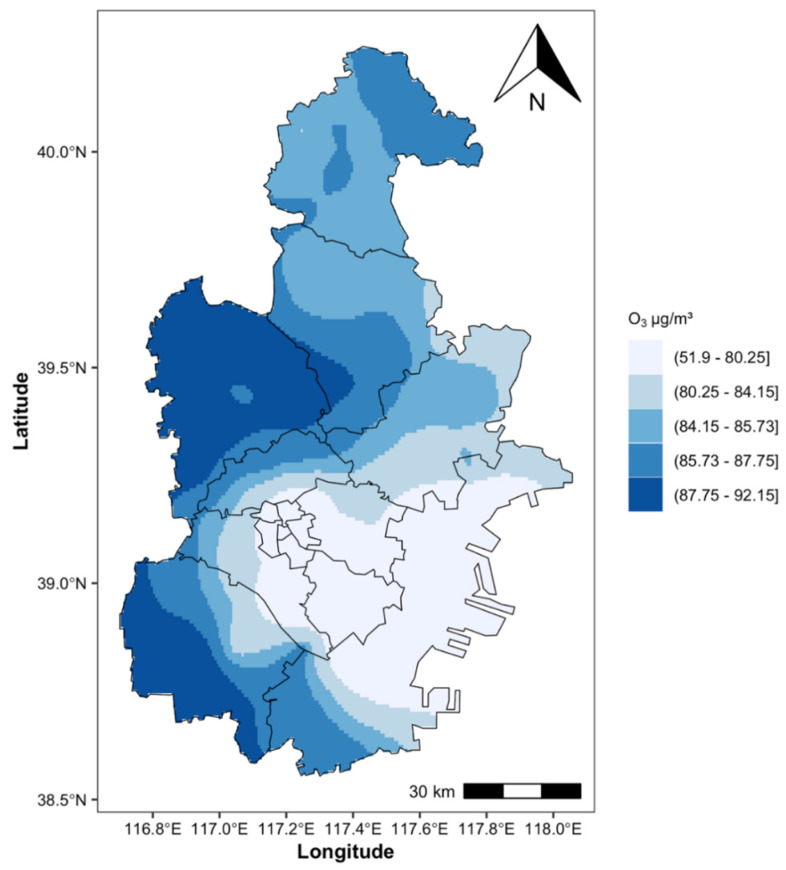
Average O_3_ concentrate study period (2017–2023) in Tianjin, China.

Participants’ residential addresses were geocoded into latitude and longitude coordinates using the Baidu Maps API (http://lbsyun.baidu.com), and the k-nearest neighbor algorithm was then used to map these geocoded addresses to the corresponding grids in the TAP dataset. We calculated the weekly-specific and trimester-specific average levels of O_3_, temperature, and dew point from 12 weeks before pregnancy to 28 weeks. The average exposure levels of O_3_ during the study period (2017–2023) are shown in Figure 1.

### 2.4. Statistical Analysis

Continuous variables were presented as mean ± standard deviation (SD), and categorical variables were expressed as frequencies and percentages (%). Independent sample *t*-tests were used to evaluate differences in continuous variables across GDM and non-GDM groups, and chi-squared tests were performed for categorical variables. Spearman correlation analysis was used to assess the correlations among O_3_, temperature, and dew point. Based on previous literature [30,36,37], covariates adjusted for in the models included: maternal age (years, continuous), preconception body mass index (BMI; <18.50, 18.50–23.99, ≥24.0 kg/m^2^), occupation (manual labor, non-manual labor and unemployed), education level (less than high school, high school, more than high school), gravidity (nulligravida, multigravida), parity (nullipara, multipara), conception season (spring: March to May, summer: June to August, autumn: September to November, winter: December to February), gestational hypertension (yes, no), and infant sex (female, male). A natural cubic spline function with 3 degrees of freedom was used to model the potential non-linear relationships of temperature and dew point with GDM.

First, logistic regression models were used to analyze the associations between average trimester-specific O_3_ exposure and GDMA1 and GDMA2. Odds ratios (ORs) and 95% confidence interval were estimated for each 10 μg/m^3^ increase in O_3_. Subsequently, distributed lag nonlinear models (DLNMs) with logistic regression model were applied to investigate the nonlinear and lagged associations between O_3_ exposures and GDMA1 and GDMA2 [38,39]. Given that our preliminary analyses confirmed significant non-linear associations between O_3_ exposure and both GDMA1 and GDMA2, the exposure–response dimension of the cross-basis function in DLNMs was modeled using natural splines, with the optimal degrees of freedom selected based on the Akaike Information Criterion (AIC). The lag-response dimension was modeled using a natural cubic spline with 5 degrees of freedom [40]. The maximum lag was set at 40 weeks (from 12 weeks before pregnancy to 28 weeks) to estimate the lagged effects of O_3_ exposure during this period and to identify potential sensitive windows. The Class II concentration limits (160 μg/m^3^) of the National Environmental Quality Standard (GB3095-2012) were used as O_3_ reference value in DLNMs [41].

To explore the effect of temperature on the association between O_3_ and GDMA1 and GDMA2, a multiplicative interaction term of temperature and O_3_ was incorporated into the models. To investigate the health impacts of extreme temperature events (e.g., heatwaves), weekly average temperature was divided into three levels using the 10th and 90th percentiles as cut-off (specific cut-offs values are provided in Table S1), consistent with previous epidemiological studies evaluating extreme temperature effects [27,42,43]. Percentile-based thresholds were used because they account for local climatic conditions and population adaptation to temperature, rather than relying on fixed absolute temperature values. Subgroup analyses were performed to identify susceptible populations based on maternal age (<35/≥35 years), preconception overweight, gravidity, parity, family history of diabetes, and gestational hypertension. Differences between strata were assessed using the likelihood ratio test [36,44].

To evaluate the robustness of the results, the following sensitivity analyses were conducted: (1) the O_3_ reference concentration in the DLNMs was changed in the models to the Class I concentration limit (100 μg/m^3^); (2) maternal smoking and drinking were additionally adjusted for in the models; (3) two-pollutant models was constructed to assess the potential confounding effect of PM_2.5_; (4) different percentile cut-offs (25th and 75th percentile; 5th and 95th percentile) were used to define temperature levels when analyzing the effect modification of temperature; (5) investigate the effects of O_3_ exposure on GDMA1 and GDMA2 between the pre-pandemic period (2017–2019) and the pandemic period (2020–2023) in Tianjin, China. All statistical analyses were performed with R software (version 4.2.0) and two-sided *p* values < 0.05 were considered statistically significant.

## 3. Results

### 3.1. Characteristics of Participants

Table 1 presents the baseline characteristics of participants in this study. Among the 11,491 pregnant women included, 2323 (20.22%) were diagnosed with GDM, comprising 1984 (17.27%) with GDMA1 and 339 (2.95%) with GDMA2. Women with GDM were older than those without GDM, with mean ages of 32.6 years in the GDMA1 group, 33.2 years in the GDMA2 group, and 31.6 years in the non-GDM group. Women with GDM had higher rates of preconception overweight or obesity (BMI ≥ 24), gestational hypertension, and large for gestational age (LGA) compared to women without GDM. Women residing in rural areas had a higher proportion of GDM (both GDMA1 and GDMA2) than those in urban areas, and conception in summer or autumn was more common among GDM cases compared with non-GDM women.

### 3.2. Distribution and Correlations of O_3_ Concentration and Meteorological Factors

Table S2 shows the distribution and correlation of O_3_ concentration and meteorological factors for trimester-specific exposure windows. The average O_3_ concentration and temperature in the preconception were slightly higher than those in other trimesters. Specifically, the average O_3_ concentrations were 105.55 μg/m^3^ (preconception), 102.93 μg/m^3^ (first trimester), 100.25 μg/m^3^ (second trimester), and 101.52 μg/m^3^ (from last menstrual period to 28 weeks of gestation), respectively. While average temperatures during the corresponding periods were 15.02 °C, 14.53 °C, 13.43 °C, and 13.95 °C. Spearman’s correlation analysis indicated a strong association between O_3_ and temperature within each trimester-specific exposure windows (r = 0.93), with a corresponding variance inflation factor of 7.4, which is moderately above the conventional threshold of 5 but well below 10. Given our primary interest in temperature as an effect modifier and the biological plausibility of their interaction, we retained both variables in the main models and further performed stratified analyses by temperature to evaluate the modification effects.

### 3.3. Associations of O_3_ Exposure with GDMA1 and GDMA2

Associations of trimester-specific O_3_ exposure with the risk of GDMA1 and GDMA2 are shown in Table 2. Without any adjustment, logistic regression analysis showed that preconception O_3_ exposure was associated with a small increase in GDMA1 risk. For each 10 μg/m^3^ increase in O_3_ concentration, the risk of GDMA1 increased by 1.7% (OR = 1.017, 95% CI: 1.006, 1.029). However, after adjusting for all covariates, O_3_ exposure showed no statistically significant association with GDMA1 and GDMA2 across any trimester-specific exposure windows.

**Figure 2:**
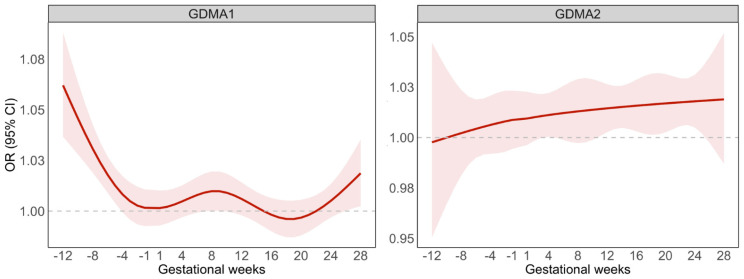
Associations of week-specific O_3_ exposure with the risk of GDMA1 and GDMA2 during preconception 12 weeks to 28 weeks of gestation. Abbreviations: GDM, gestational diabetes mellitus; GDMA1, diet-controlled GDM; GDMA2, insulin-treated GDM; OR, odds ratio; 95% CI, 95% confidence interval. Note: Distributed lag non-linear models (DLNMs) incorporated with logistic regression was used to calculate adjusted ORs (95% CIs) each 10 μg/m^3^ increment in the concentrations of O_3_ at a weekly level over the preconception period and pregnancy. All models adjusted for maternal age, preconception BMI, educational level, occupation, gravidity, parity, conception season, gestational hypertension, newborn gender, and natural cubic splines with 3 degrees of freedom for ambient temperature and dew point. The red shadow indicate 95% confidence intervals.

To further identify the sensitive exposure gestational weeks, we applied DLNMs to examine the weekly-specific associations of O_3_ exposure with GDMA1 and GDMA2 from 12 weeks before pregnancy to 28 weeks of gestation. The results are shown in Figure 2. For each 10 μg/m^3^ increase in O_3_ was statistically associated with a small increase in GDMA1 risk during 5–12 weeks before pregnancy and 26–28 weeks of gestation, with the largest effects observed at 12 weeks before pregnancy (OR = 1.062, 95% CI: 1.037, 1.088) and 28 weeks of gestation (OR = 1.019, 95% CI: 1.002, 1.035) (Table S3). The association gradually weakened as the exposure window moved closer to conception. In contrast, weak associations with GDMA2 were observed during 11–25 weeks of gestation, with the largest estimate at 25th weeks of gestation (OR = 1.018, 95% CI: 1.002, 1.035) (Table S3).

### 3.4. Modification Effect of Ambient Temperature in O_3_ Exposure with GDMA1 and GDMA2

**Figure 3:**
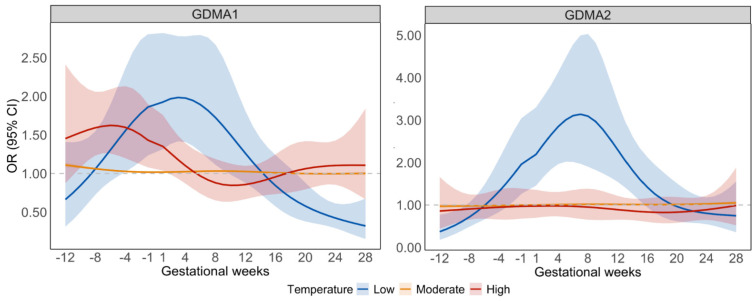
Modification effect of ambient temperature on the association of O_3_ exposure with the risk of GDMA1 and GDMA2. Abbreviations: GDM, gestational diabetes mellitus; GDMA1, diet-controlled GDM; GDMA2, insulin-treated GDM; OR, odds ratio; 95% CI, 95% confidence interval. Note: Weekly average temperature was divided into three levels using the 10th and 90th percentiles. Distributed lag non-linear models (DLNMs) incorporated with logistic regression was used to calculate adjusted ORs (95% CIs) each 10 μg/m^3^ increment in the concentrations of O_3_ at a weekly level over the preconception period and pregnancy. All models adjusted for maternal age, preconception BMI, educational level, occupation, gravidity, parity, conception season, gestational hypertension, newborn gender, and natural cubic splines with 3 degrees of freedom for dew point.

Figure 3 presents the modification effect of ambient temperature in O_3_ exposure with GDMA1 and GDMA2. The observed association of O_3_ on GDMA1 increased with rising temperature in 1–9 weeks before pregnancy (*p* for interaction < 0.05), with the largest effect modification observed at 6 weeks before pregnancy (Table S4). Specifically, under high temperature conditions (>90th percentiles), for every 10 μg/m^3^ increase in O_3_ concentration at 6 weeks before pregnancy, the risk of GDMA1 increased by 62.2% (OR = 1.622, 95% CI: 1.259, 2.091) (Table S4). Conversely, O_3_ exposure during the 1–15 weeks of pregnancy was positively associated with GDMA2 under low temperature conditions (<10th percentiles) (*p* for interaction < 0.05).

### 3.5. Subgroup Analysis

**Figure 4:**
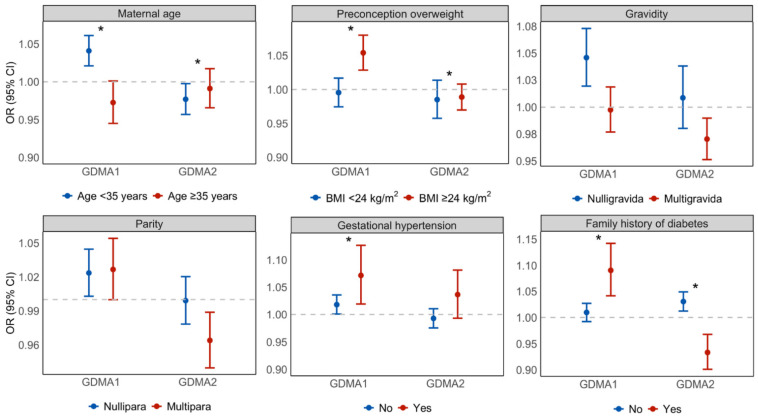
Subgroup analysis for the association of O_3_ exposure with the risk of GDMA1 and GDMA2. Abbreviations: GDM, gestational diabetes mellitus; GDMA1, diet-controlled GDM; GDMA2, insulin-treated GDM; OR, odds ratio; 95% CI, 95% confidence interval. Note: Results at the most sensitive (with the highest effect estimates) windows of O_3_ exposure for GDMA1 (28th week after pregnancy) and GDMA2 (25th week after pregnancy) were selected for result presentation. All models adjusted for maternal age, preconception BMI, educational level, occupation, gravidity, parity, conception season, gestational hypertension, newborn gender, and natural cubic splines with 3 degrees of freedom for ambient temperature and dew point (except for the stratification variables). * *p* for interaction < 0.05. The dashed line is the reference line at OR = 1.

Subgroup analysis for the association of O_3_ exposure with the risk of GDMA1 and GDMA2 within the most sensitive windows are shown in Figure 4. The results indicated that maternal age, preconception obesity, gestational hypertension, and family history of diabetes may modify the association between preconception and gestational O_3_ and GDMA1 (*p* for interaction < 0.05) (Table S5). Specifically, each 10 μg/m^3^ increase in O_3_ at 28 weeks after pregnancy was associated with small increases in the odds of GDMA1 among women aged <35 years (OR = 1.041, 95% CI: 1.021, 1.061), those with preconception overweight (OR = 1.054, 95% CI: 1.029, 1.080), those with gestational hypertension (OR = 1.071, 95% CI: 1.019, 1.126), and those with family history of diabetes (OR = 1.091, 95% CI: 1.042, 1.142) (Table S5). Conversely, each 10 μg/m^3^ increase in O_3_ at 25 weeks of gestation was associated with an increased risk of GDMA2 in women without family history of diabetes (OR = 1.031, 95% CI: 1.012, 1.049) (Table S5).

### 3.6. Sensitivity Analysis

Sensitivity analyses indicated that the main findings were generally robust (Tables S6–S10). After adjusting the reference concentration of O_3_ in the DLNMs to the primary concentration limit (100 μg/m^3^), the association between O_3_ exposure and GDMA1 weakened during few gestational weeks, while its effect on GDMA2 remained broadly consistent (Table S6). When additionally adjusting for maternal smoking and drinking or including PM_2.5_ to construct a two-pollutant model, the associations of preconception and gestational O_3_ exposure with GDMA1 and GDMA2 remained consistent, with the most sensitive gestational weeks aligning with the main results (Tables S7 and S8). When temperature was categorized using different percentile cut-offs, with 25th and 75th percentile as the cut-off, the adverse effect of O_3_ on GDMA1 was only observed to increase with rising temperature during gestation but not preconception (Table S9). With 5th and 95th percentile as the cut-off, the modification effect of ambient temperature on the association between O_3_ exposure and the risk of GDMA1 remained generally consistent with the main results (Table S10). Compared with the pre-COVID-19 pandemic period, ambient O_3_ concentrations decreased during the pandemic. The effect estimates of O_3_ exposure on GDMA1 and GDMA2 were attenuated in the pandemic period. No statistically significant association was found between O_3_ exposure and GDMA1, whereas weak but statistically significant associations remained for GDMA2 during the 11–25 weeks of gestation window (Table S11).

## 4. Discussion

In this retrospective cohort study of 11,491 Chinese pregnant women, we evaluated the associations between preconception and gestational ambient O_3_ exposure and different clinical classifications of GDM and explored the effect modification by ambient temperature. We observed 5–12 weeks before pregnancy and 26–28 gestational weeks as sensitive windows where O_3_ exposure was weakly and statistically associated with small increases in GDMA1 risk, and 11–25 gestational weeks for GDMA2. However, no statistical association was observed between trimester-specific O_3_ exposure and GDMA1 or GDMA2. Furthermore, ambient temperature appeared to modify the association between O_3_ exposure and GDMA1, with high temperatures strengthening the observed association between preconception O_3_ exposure on GDMA1. Additionally, we found that gestational O_3_ exposure was associated with a higher risk of GDMA1 among women under 35 years of age, preconception obesity, gestational hypertension, and family history of diabetes.

Our results showed that preconception O_3_ exposure was associated with an increased risk of GDMA1 when the model was unadjusted. However, no statistical associations were observed for any trimester-specific after full adjustment for covariates. Currently, several studies have investigated the relationship between O_3_ exposure and GDM, but their results and identified sensitive windows are inconsistent. For instance, two cohort studies conducted in China reported that the preconception and first-trimester were sensitive windows associated with GDM [17,19], while Zhang et al. reported increased GDM risk associated with O_3_ exposure during the first two trimesters [10]. Evidence from large US cohort studies has also been inconsistent. One electronic medical record-based cohort study reported that O_3_ exposure appeared to increase GDM risk in association with second trimester exposure, but not during preconception or earlier pregnancy windows [14]. In contrast, another large US cohort study reported inverse associations between preconception and first trimester O_3_ exposure and GDM risk [15]. However, a cohort study by Yan et al. in China reported no significant statistical association between O_3_ exposure and GDM across different trimester-specific periods in the single-pollutant model [45], which is consistent with our trimester-specific findings. Although that study also reported significant associations in two-pollutant models involving PM_2.5_ and gaseous pollutants, suggesting that the air pollution–GDM relationship may be influenced by multipollutant exposure patterns and model specifications. The discrepant findings between our study and previous research may be related to two key factors. First, unlike most studies that treat GDM as a homogeneous disease outcome, our investigation distinguished between clinical classifications (GDMA1 and GDMA2), suggesting potential differential effects of O_3_ exposure according to GDM severity. Second, although our study included 11,491 pregnant women, the statistical power remained limited when analyzing GDM classifications, particularly for the less prevalent GDMA2 group (2.95%). Future studies with larger sample sizes and refined disease classifications are therefore warranted to obtain more robust and precise estimates.

We further employed DLNM combined with logistic regression to identify susceptible gestational weeks to O_3_ exposure in pregnant women. DLNM suggested 5–12 weeks preconception and 26–28 gestational weeks as sensitive windows for increased GDMA1 risk, and 11–25 gestational weeks for GDMA2. Compared with conventional logistic regression, DLNMs more accurately captured exposure response relationships and yielded distinct results, likely because traditional models fail to account for the complex lag patterns of O_3_ effects and are susceptible to multicollinearity in exposure data [38]. Studies applying DLNMs to investigate sensitive windows for O_3_ exposure and GDM remain limited [10,18,46]. A Canadian cohort study reported that O_3_ exposure during 1–10 weeks preconception and 9–28 gestational weeks was associated with increased GDM risk [46], which aligns closely with our findings. Given these findings, future studies should adopt DLNMs to further validate weekly-specific associations between O_3_ exposure and GDM classifications. Targeted interventions during identified sensitive windows and development of staged, personalized O_3_ protection strategies for susceptible populations are warranted. Furthermore, although the observed ORs per 10 μg/m^3^ increase in O_3_ exposure were statistically significant, their magnitudes were small. Therefore, these findings should be interpreted cautiously, as they may be susceptible to statistical noise, residual confounding, and exposure measurement error. Rather than indicating a strong individual-level effect, these results suggest a weak association that requires confirmation in future studies.

The precise mechanisms underlying the association between O_3_ exposure and the development of GDM have not been elucidated. Current evidence suggests that O_3_ may contribute to the disease process through multiple pathways. As a potent oxidizing pollutant, O_3_ may induce the generation of substantial reactive oxygen species (ROS) in the lungs, thereby potentially triggering oxidative stress and contributing to hepatic insulin resistance [47,48]. Experimental evidence suggests that O_3_ exposure may impair glucose metabolism by inducing oxidative stress and endoplasmic reticulum stress in skeletal muscle, followed by activation of c-Jun N-terminal kinase (JNK). This pathway may disrupt insulin signaling and contribute to insulin resistance [10,48]. This pathological cascade may provide a possible biological explanation for GDMA1, characterized predominantly by insulin resistance with preserved pancreatic β-cell function [49]. The persistence of this state may further compromise β-cell function through systemic inflammation and oxidative stress, potentially limiting insulin secretion and thereby contributing to inadequate compensation for the rising insulin demands of pregnancy, which may promote progression to the insulin-requiring GDMA2 [9,50]. Therefore, further molecular and cellular studies are urgently needed to validate these hypothesized mechanisms and elucidate the impact of O_3_ exposure on different GDM classifications.

Notably, our study suggests that ambient temperature, especially high temperature may modify the association between O_3_ exposure and the risk of GDMA1. For a given level of O_3_ exposure, higher preconception temperature was associated with a significantly increased risk of GDMA1. Consistently, a cohort study conducted in China reported that O_3_ exposure during the preconception and first trimester was associated with an elevated risk of GDM in warm seasons but not in cold seasons. Although that study adjusted for temperature only as a confounder without evaluating its potential effect modification [19]. Several biological pathways may underlie the joint effects of O_3_ and high temperature. Pregnancy is especially susceptible to O_3_-induced inflammatory responses, given its reliance on immune tolerance, vascular adaptation, and placental function [51,52]. High temperatures may enhance the uptake and tissue penetration of O_3_ by increasing respiratory rate, promoting vasodilation, and altering blood flow velocity [53]. And heat stress itself may act synergistically with O_3_-induced oxidative stress, potentially exacerbating insulin resistance [54,55]. Ambient O_3_-related and temperature-modified adverse reproductive and pregnancy outcomes may differ by exposure window because the vulnerable biological systems change across reproductive stages. Biologically, the preconception period represents a critical window for oocyte maturation and embryo implantation. O_3_-induced oxidative stress and systemic inflammation may impair gamete quality or disrupt early epigenetic programming, thereby contributing to subsequent metabolic dysfunction during pregnancy [56,57]. During the second trimester, elevated maternal basal metabolic rate and accelerated fetal growth exacerbate glucose metabolic disorders and insulin resistance, potentially enhancing susceptibility to environmental exposures [58]. This timing-specific pattern is consistent with our finding that temperature modification was most pronounced for preconception O_3_ exposure and GDMA1, rather than uniformly across all gestational windows or GDM classifications.

Global climate change is expected to increase the frequency, intensity and duration of extreme weather events (such as heatwaves), rising temperatures may intensify the adverse health effects of O_3_ exposure [26]. This potential synergistic effect between high temperature and O_3_ suggests that more stringent air quality standards may be necessary in warmer climates or during warm seasons to ensure adequate public health protection. Furthermore, pregnant women should be reducing outdoor activities during periods of concurrently high temperature and O_3_ concentration to lower exposure risks and related adverse health outcomes.

Our analysis identified that pregnant women under 35 years of age, with preconception obesity, gestational hypertension, and family history of diabetes appeared more susceptible to increased risk of GDMA1 when exposed to O_3_. Contrary to the conventional understanding that advanced maternal age represents an independent risk factor for GDM, we observed that younger women (<35 years) showed a higher susceptibility to O_3_-associated GDMA1 than older individuals. Age-related heterogeneity in the association between air pollution and GDM has been reported in previous study. For example, a Massachusetts cohort study found that second trimester PM_2.5_ exposure was associated with GDM only in the youngest maternal age stratum [59]. Our finding may reflect potential age-related susceptibility or differences in baseline metabolic risk, although the underlying mechanism remains uncertain and should be further examined in studies with detailed behavioral and biological data. O_3_ may act synergistically with pre-existing chronic low-grade inflammation and elevated adipokines to promote insulin resistance in women with preconception obesity [60]. Among those with gestational hypertension, O_3_-induced oxidative stress could exacerbate endothelial dysfunction, thereby potentially impairing insulin signaling and glucose homeostasis [61,62]. Furthermore, pregnant women with family history of diabetes may carry genetic susceptibility genes and may have potential defects such as insufficient pancreatic β-cell function reserve or reduced insulin sensitivity [4,63]. O_3_ may act as an environmental stressor that could accelerate the transition from prediabetes to GDM. Inversely, we observed a negative association between O_3_ exposure and GDMA2 in pregnant women with a family history of diabetes. This sign reversal lacks a clear biological explanation and may reflect unstable subgroup estimates, residual confounding, exposure measurement error, or unmeasured differences in clinical management. Women with GDMA2 generally may require pharmacotherapy because glycemic targets are not achieved with lifestyle management, and more intensive antenatal surveillance [3,64]. However, information on glycemic management intensity, antenatal visit frequency, and post-diagnosis behavioral changes was unavailable in our dataset. Future studies should integrate ambient pollution monitoring with lifestyle data to develop targeted O_3_ protective guidelines for high-risk pregnant women.

This study has several notable strengths. First, to our knowledge, it is the first to examine the associations between preconception and gestational O_3_ exposure and distinct clinical classifications of GDM. Second, we investigated the modifying effect of ambient temperature on the O_3_–GDM classifications associations, suggesting that high temperatures may amplify the adverse effect of preconception O_3_ exposure on GDMA1 risk. Third, we used DLNMs to assess week-specific O_3_ effects on GDMA1 and GDMA2, thereby identifying non-linear exposure response relationships and critical windows. Finally, we identified subgroups that were particularly susceptible to O_3_ exposure, including woman under 35 years of age, those with preconception obesity, gestational hypertension, and family history of diabetes, underscoring the necessity of targeted interventions for vulnerable populations.

Several limitations of this study should also be mentioned. First, the relatively low incidence of GDMA2 limited the statistical power, particularly in subgroup analyses. In addition, because multiple weekly-specific and stratified estimates were examined, and no formal multiple comparison correction was applied, some statistically significant findings may have occurred by chance and should be interpreted cautiously. Second, as a single-center study, the generalizability of our findings to other regions or countries may be limited, as environmental conditions (e.g., O_3_ levels and climate patterns), healthcare practices, and GDM screening/management protocols may differ substantially across populations. Third, the spatial resolution of O_3_ exposure (10 km) may not adequately capture finer-scale concentration variations, and the lack of indoor O_3_ measurements or individual activity patterns (such as commuting behavior and time spent indoors) further limited the precision of individual exposure assessment. Finally, several potentially important factors, including genetic susceptibility, dietary habits, and geographical variations, may influence the risk of GDM and potentially modify the association between ozone exposure and GDM. However, due to the lack of detailed individual-level genetic and dietary information, as well as limited geographical data, we were unable to further assess these factors in the present study. Future studies with more comprehensive individual and contextual information are warranted to clarify their potential roles.

## 5. Conclusions

In summary, this study did not find statistically significant associations in the fully adjusted trimester-specific models, but the weekly-specific DLNM analyses suggested weak associations between maternal O_3_ exposure and GDMA1 and GDMA2 during several potential sensitive windows. High temperatures appeared to strengthen the observed association between preconception O_3_ exposure and GDMA1. Given the small effect sizes in several weekly specific estimates, these findings should be interpreted cautiously and validated in future studies. These results suggest that O_3_ exposure protection may need to consider different clinical classifications and potential sensitive windows, while the modifying role of ambient temperature may be considered in health risk assessments of O_3_-related GDM.

## Figures and Tables

**Table 1:** Descriptive characteristics of the study population.

Characteristics	All (*n* = 11,491)	Non-GDM (*n* = 9168)	GDM (*n* = 2323)	GDMA1 (*n* = 1984)	GDMA2 (*n* = 339)	*p*
Maternal age, years, mean (SD)	31.8 (4.2)	31.6 (4.2)	32.7 (4.1)	32.6 (4.1)	33.2 (4.3)	<0.001
Preconception BMI, kg/m^2^, *n* (%)						<0.001
<18.50	1094 (9.5)	1014 (11.1)	80 (3.4)	76 (3.8)	4 (1.2)	
18.50–23.99	6913 (60.2)	5793 (63.2)	1120 (48.2)	1017 (51.3)	103 (30.4)	
≥24	3484 (30.3)	2361 (25.7)	1123 (48.4)	891 (44.9)	232 (68.4)	
Maternal educational level, *n* (%)						<0.001
Less than high school	383 (3.3)	278 (3.0)	105 (4.5)	88 (4.4)	17 (5.0)	
High school	603 (5.2)	419 (4.6)	184 (7.9)	143 (7.2)	41 (12.1)	
More than high school	9832 (85.6)	7960 (86.8)	1872 (80.6)	1615 (81.4)	257 (75.8)	
Missing	673 (5.9)	511 (5.6)	162 (7)	138 (7.0)	24 (7.1)	
Maternal occupation, *n* (%)						<0.001
Manual worker	426 (3.7)	322 (3.5)	104 (4.5)	83 (4.2)	21 (6.2)	
Non-manual workers and unemployed	10,002 (87.0)	8042 (87.7)	1960 (84.4)	1671 (84.2)	289 (85.3)	
Missing	1063 (9.3)	804 (8.8)	259 (11.1)	230 (11.6)	29 (8.6)	
Maternal residence, *n* (%)						0.005
Urban	7361 (64.1)	5932 (64.7)	1429 (61.5)	1231 (62.0)	198 (58.4)	
Rural	4130 (35.9)	3236 (35.3)	894 (38.5)	753 (38.0)	141 (41.6)	
Conception season, *n* (%)						0.011
Spring (March to May)	2764 (24.1)	2213 (24.1)	551 (23.7)	470 (23.7)	81 (23.9)	
Summer (June to August)	3148 (27.3)	2481 (27.1)	667 (28.7)	578 (29.1)	89 (26.3)	
Autumn (September to November)	3005 (26.2)	2366 (25.8)	639 (27.5)	541 (27.3)	98 (28.9)	
Winter (December to February)	2574 (22.4)	2108 (23.0)	466 (20.1)	395 (19.9)	71 (20.9)	
Gravidity, *n* (%)						<0.001
Primigravidae	5377 (46.8)	4383 (47.8)	994 (42.8)	866 (43.6)	128 (37.8)	
Multigravida	6114 (53.2)	4785 (52.2)	1329 (57.2)	1118 (56.4)	211 (62.2)	
Parity, *n* (%)						0.164
Primiparity	7808 (67.9)	6258 (68.3)	1550 (66.7)	1330 (67.0)	220 (64.9)	
Multiparity	3683 (32.1)	2910 (31.7)	773 (33.3)	654 (33.0)	119 (35.1)	
Maternal smoking, *n* (%)						<0.001
Yes	109 (0.9)	72 (0.8)	37 (1.6)	26 (1.3)	11 (3.2)	
No	11,346 (98.8)	9060 (98.8)	2286 (98.4)	1958 (98.7)	328 (96.8)	
Missing	36 (0.3)	36 (0.4)	0	0	0	
Maternal drinking, *n* (%)						0.010
Yes	33 (0.3)	25 (0.3)	8 (0.3)	5 (0.3)	3 (0.9)	
No	11,422 (99.4)	9107 (99.3)	2315 (99.7)	1979 (99.7)	336 (99.1)	
Missing	36 (0.3)	36 (0.4)	0	0	0	
Gestational hypertension, *n* (%)						<0.001
Yes	914 (8.0)	635 (6.9)	279 (12)	231 (11.6)	48 (14.2)	
No	10,577 (92.0)	8533 (93.1)	2044 (88)	1753 (88.4)	291 (85.8)	
LGA, *n* (%)						<0.001
Yes	1401 (12.2)	988 (10.8)	413 (17.8)	329 (16.6)	84 (24.8)	
No	10,090 (87.8)	8180 (89.2)	1910 (82.2)	1655 (83.4)	255 (75.2)	
Gestational age, weeks, mean (SD)	38.5 (1.9)	38.6 (1.9)	38.3 (1.8)	38.3 (1.9)	38.2 (1.4)	0.134
Newborn gender, *n* (%)						<0.001
Male	5871 (51.1)	4624 (50.4)	1247 (53.6)	1069 (53.9)	178 (52.5)	
Female	5439 (47.3)	4364 (47.6)	1075 (46.3)	914 (46.1)	161 (47.5)	
Missing	181 (1.6)	180 (2)	1 (0.1)	1 (0.1)	0	
Birth weight, g, mean (SD)	3300.8 (564.0)	3288.5 (555.5)	3349.1 (594.3)	3337 (597.8)	3419.7 (569.4)	<0.001

Abbreviations: GDM, gestational diabetes mellitus; GDMA1, diet-controlled GDM; GDMA2, insulin-treated GDM; SD, standard deviation; BMI, body mass index; LGA, large for gestational age.

**Table 2:** Associations of trimester-specific O_3_ exposure with the risk of GDMA1 and GDMA2.

Exposure	GDMA1		GDMA2	
	Crude OR (95% CI)	Adjusted OR (95% CI)	Crude OR (95% CI)	Adjusted OR (95% CI)
Preconception	**1.017 (1.006, 1.029)**	0.980 (0.923, 1.042)	1.009 (0.984, 1.036)	1.119 (0.977, 1.282)
First trimester	0.999 (0.987, 1.010)	0.959 (0.906, 1.016)	0.995 (0.970, 1.021)	0.942 (0.829, 1.069)
Second trimester	0.978 (0.966, 0.990)	1.031 (0.973, 1.093)	0.995 (0.969, 1.022)	0.926 (0.815, 1.053)
LMP to 28 weeks’ gestation	0.973 (0.956, 0.990)	1.002 (0.916, 1.096)	0.990 (0.952, 1.029)	0.850 (0.698, 1.036)

Abbreviations: GDM, gestational diabetes mellitus; GDMA1, diet-controlled GDM; GDMA2, insulin-treated GDM; OR, odds ratio; 95% CI, 95% confidence interval; LMP, last menstrual period. Note: Logistic regression was used to calculate adjusted ORs (95% CIs) each 10 μg/m^3^ increment in the concentrations of O_3_ at a weekly level over the preconception period and pregnancy. All models adjusted for maternal age, preconception BMI, educational level, occupation, gravidity, parity, conception season, gestational hypertension, newborn gender, and natural cubic splines with 3 degrees of freedom for ambient temperature and dew point. Black bold indicate statistically significance (*p* < 0.05) with positive effects.

## Data Availability

The raw data supporting the conclusions of this article will be made available by the authors on request.

## Supplementary Materials

The following supporting information can be downloaded at: https://www.mdpi.com/article/10.3390/toxics14070622/s1, Figure S1: The inclusion and exclusion process of the study population; Table S1: Weekly ambient temperature cutoffs from 12 weeks before pregnancy to 28 weeks of gestation; Table S2: Distribution and correlations of O_3_ and meteorological variables at different exposure windows; Table S3: Associations of week-specific O_3_ exposure with the risk of GDMA1 and GDMA2 from 12 weeks before pregnancy to 28 weeks of gestation; Table S4: Modification effect of ambient temperature on the association of O_3_ exposure with the risk of GDMA1 and GDMA2; Table S5: Subgroup analysis for the association of O_3_ exposure with the risk of GDMA1 and GDMA2 within the most sensitive preconception and pregnancy exposure windows; Table S6: Sensitivity analysis of the association of O_3_ exposure with the risk of GDMA1 and GDMA2 using the Class I concentration limits (100 μg/m^3^) of GB3095-2012 as the reference in DLNM models; Table S7: Sensitivity analysis of the association of O_3_ exposure with the risk of GDMA1 and GDMA2, with further adjustments of maternal smoking and drinking covariates; Table S8: Sensitivity analysis of the association of O_3_ exposure with the risk of GDMA1 and GDMA2 in two-pollutant models; Table S9: Sensitivity analysis of modification effect of ambient temperature (stratified by the 25th and 75th percentiles) on the association of O_3_ exposure with the risk of GDMA1 and GDMA2; Table S10: Sensitivity analysis of modification effect of ambient temperature (stratified by the 5th and 95th percentiles) on the association of O_3_ exposure with the risk of GDMA1 and GDMA2; Table S11: Sensitivity analysis of effects of O_3_ exposure on GDMA1 and GDMA2 between the pre-pandemic period (2017–2019) and the pandemic period (2020–2023).
