# Supplementary material for: Effect Modification by Ambient Temperature on the Association of Ambient Ozone Exposure with Diet-Controlled and Insulin-Treated Gestational Diabetes Mellitus
Source: Toxics. 2026 Jul 16;14(7):622. doi: 10.3390/toxics14070622 (PMC13417651; doi:10.3390/toxics14070622)
Supplement: Supplementary file 1 [file toxics-14-00622-s001.zip › toxics-4383712-supplementary.pdf]

Supplemental Materials

Effect Modification by Ambient Temperature on the Association of Ambient Ozone Exposure with Diet-Controlled and Insulin-Treated Gestational Diabetes Mellitus

Table of Contents

Table S1. Weekly ambient temperature cutoffs from 12 weeks before pregnancy to 28 weeks of gestation

Table S2. Distribution and correlations of O_3 and meteorological variables at different exposure windows

Table S3. Associations of week-specific O_3 exposure with the risk of GDMA1 and GDMA2 from 12 weeks before pregnancy to 28 weeks of gestation

Table S4. Modification effect of ambient temperature on the association of O_3 exposure with the risk of GDMA1 and GDMA2

Table S5. Subgroup analysis for the association of O_3 exposure with the risk of GDMA1 and GDMA2 within the most sensitive preconception and pregnancy exposure windows

Table S6. Sensitivity analysis of the association of O_3 exposure with the risk of GDMA1 and GDMA2 using the Class I concentration limits (100 μg/m^3) of GB3095-2012 as the reference in DLNM models

Table S7. Sensitivity analysis of the association of O_3 exposure with the risk of GDMA1 and GDMA2, with further adjustments of maternal smoking and drinking covariates

Table S8. Sensitivity analysis of the association of O_3 exposure with the risk of GDMA1 and GDMA2 in two-pollutant models

Table S9. Sensitivity analysis of modification effect of ambient temperature (stratified by the 25th and 75th percentiles) on the association of O_3 exposure with the risk of GDMA1 and GDMA2

Table S10. Sensitivity analysis of modification effect of ambient temperature (stratified by the 5th and 95th percentiles) on the association of O_3 exposure with the risk of GDMA1 and GDMA2

Table S11. Sensitivity analysis of effects of O_3 exposure on GDMA1 and GDMA2 between the pre-pandemic period (2017–2019) and the pandemic period (2020–2023).

Figure of Contents

Figure S1. The inclusion and exclusion process of the study population

Table S1. Weekly ambient temperature (°C) cutoffs from 12 weeks preconception to 28 weeks of pregnancy

Exposure	Different cutoffs					
	5th percentile	10th percentile	25th percentile	75th percentile	90th percentile	95th percentile
**Preconception**						
12 Weeks	-2.58	-0.89	4.49	25.27	27.79	28.97
11 Weeks	-2.60	-0.94	4.81	25.29	27.74	28.94
10 Weeks	-2.71	-0.97	4.81	25.31	27.70	28.86
9 Weeks	-2.71	-1.07	4.89	25.26	27.70	28.84
8 Weeks	-2.49	-0.94	4.94	25.22	27.70	28.92
7 Weeks	-2.60	-0.96	4.91	25.22	27.59	28.82
6 Weeks	-2.71	-1.08	4.81	25.20	27.55	28.85
5 Weeks	-2.71	-1.07	4.89	25.23	27.55	28.78
4 Weeks	-2.79	-1.00	4.85	25.24	27.60	28.80
3 Weeks	-2.69	-1.15	4.80	25.20	27.54	28.85
2 Weeks	-2.70	-1.08	4.81	25.19	27.57	28.87
1 Weeks	-2.62	-1.07	4.86	25.23	27.69	28.84
**After pregnancy**						
1 Weeks	-2.63	-1	4.85	25.2	27.67	28.91
2 Weeks	-2.71	-1.07	4.81	25.27	27.67	28.82
3 Weeks	-2.7	-1.08	4.85	25.25	27.69	28.87
4 Weeks	-2.6	-0.96	4.81	25.25	27.8	29.01
5 Weeks	-2.71	-0.97	4.8	25.25	27.69	28.94
6 Weeks	-2.68	-1.05	4.79	25.21	27.76	28.91
7 Weeks	-2.7	-0.96	4.79	25.25	27.73	28.97
8 Weeks	-2.71	-1.12	4.39	25.21	27.67	28.87
9 Weeks	-2.79	-1.12	4.08	25.16	27.55	28.82
10 Weeks	-2.7	-1.07	3.64	25.01	27.55	28.77
11 Weeks	-2.74	-1.32	3.53	24.93	27.48	28.77
12 Weeks	-2.89	-1.3	3.42	24.8	27.48	28.69
13 Weeks	-2.79	-1.54	3.06	24.78	27.38	28.49
14 Weeks	-2.74	-1.54	3.04	24.62	27.34	28.49
15 Weeks	-2.74	-1.71	2.71	24.57	27.28	28.49
16 Weeks	-2.71	-1.65	2.66	24.57	27.27	28.46
17 Weeks	-2.91	-1.77	2.49	24.34	27.21	28.46
18 Weeks	-2.79	-1.81	2.33	24.27	27.27	28.44
19 Weeks	-2.83	-1.7	2.49	24.34	27.3	28.46
20 Weeks	-2.89	-1.66	2.69	24.27	27.3	28.5
21 Weeks	-2.81	-1.62	2.76	24.33	27.31	28.46
22 Weeks	-2.81	-1.77	2.77	24.32	27.3	28.46
23 Weeks	-2.79	-1.62	2.84	24.27	27.3	28.5
24 Weeks	-2.7	-1.66	2.66	24.3	27.28	28.46

25 Weeks	-2.74	-1.66	2.8	24.34	27.28	28.46
26 Weeks	-2.83	-1.7	2.7	24.24	27.31	28.5
27 Weeks	-3.05	-1.9	2.7	24.24	27.34	28.53
28 Weeks	-2.91	-1.71	2.66	24.33	27.3	28.5

Table S2. Distribution and correlations of ozone and meteorological variables at different exposure windows

Exposure	Mean	SD	25th	Median	75th	IQR	O_3	$PM_{2.5}$	Temperature	Dew
**Preconception**										
O_3	105.55	41.66	64.86	110.00	142.63	77.77	1.00			
$PM_{2.5}$	51.93	16.40	38.61	50.68	65.16	26.55	-0.76	1.00		
Temperature	15.02	9.87	5.57	17.00	24.56	18.99	0.93	-0.80	1.00	
Dew point	4.51	11.57	-5.85	4.71	15.87	21.71	0.87	-0.82	0.98	1.00
**First trimester**										
O_3	102.93	41.85	62.19	102.31	141.85	79.65	1.00			
$PM_{2.5}$	51.17	15.80	38.47	49.96	64.02	25.56	-0.75	1.00		
Temperature	14.53	9.76	5.23	15.63	24.22	18.99	0.93	-0.80	1.00	
Dew point	4.31	11.47	-6.02	4.18	15.68	21.70	0.86	-0.82	0.98	1.00
**Second trimester**										
O_3	100.25	40.85	62.40	94.90	137.21	74.81	1.00			
$PM_{2.5}$	50.78	15.43	38.04	50.13	63.47	25.43	-0.76	1.00	-0.81	-0.84
Temperature	13.43	9.36	4.29	13.31	22.62	18.33	0.93	-0.81	1.00	0.98
Dew point	3.05	10.83	-6.89	2.16	13.55	20.44	0.86	-0.84	0.98	1.00
**LMP to 28 weeks' gestation**										
O_3	101.52	27.75	74.45	99.85	125.78	51.33	1.00			
$PM_{2.5}$	50.96	11.53	41.75	50.42	60.59	18.84	-0.65	1.00		
Temperature	13.95	6.32	7.54	13.80	20.15	12.61	0.93	-0.73	1.00	
Dew point	3.64	7.35	-3.55	3.83	10.86	14.41	0.85	-0.78	0.97	1.00

Abbreviations: SD, standard deviation; IQR, interquartile range; O_3, ozone; $PM_{2.5}$, fine particulate matter; LMP, last menstrual period; VIF, variance inflation factor

Table S3. Associations of week-specific O_3 exposure with the risk of GDMA1 and GDMA2 during preconception 12 weeks to 28 weeks of gestation

Exposure	GDMA1<br>Adjusted OR (95% CI)	GDMA2<br>Adjusted OR (95% CI)
**Preconception**		
12 Weeks	**1.062 (1.037, 1.088)**	0.998 (0.951, 1.047)
11 Weeks	**1.054 (1.032, 1.076)**	0.999 (0.959, 1.040)
10 Weeks	**1.046 (1.028, 1.064)**	1.000 (0.967, 1.034)
9 Weeks	**1.038 (1.024, 1.053)**	1.001 (0.975, 1.028)
8 Weeks	**1.031 (1.019, 1.043)**	1.002 (0.981, 1.024)
7 Weeks	**1.024 (1.014, 1.034)**	1.003 (0.986, 1.020)
6 Weeks	**1.018 (1.009, 1.027)**	1.004 (0.990, 1.019)
5 Weeks	**1.013 (1.004, 1.021)**	1.005 (0.992, 1.019)
4 Weeks	1.008 (0.999, 1.017)	1.006 (0.992, 1.021)
3 Weeks	1.005 (0.996, 1.014)	1.007 (0.992, 1.022)
2 Weeks	1.003 (0.993, 1.012)	1.008 (0.993, 1.023)
1 Weeks	1.002 (0.993, 1.011)	1.009 (0.994, 1.023)
**After pregnancy**		
1 Weeks	1.001 (0.993, 1.010)	1.009 (0.996, 1.023)
2 Weeks	1.002 (0.994, 1.010)	1.010 (0.998, 1.022)
3 Weeks	1.003 (0.995, 1.011)	1.011 (1.000, 1.022)
4 Weeks	1.005 (0.997, 1.013)	**1.011 (1.000, 1.022)**
5 Weeks	1.006 (0.998, 1.015)	1.012 (1.000, 1.024)
6 Weeks	1.008 (0.999, 1.017)	1.012 (0.999, 1.026)
7 Weeks	1.009 (0.999, 1.018)	1.013 (0.998, 1.028)
8 Weeks	1.010 (0.999, 1.020)	1.013 (0.997, 1.029)
9 Weeks	1.010 (0.999, 1.020)	1.013 (0.998, 1.029)
10 Weeks	1.009 (0.999, 1.018)	1.014 (0.999, 1.029)
11 Weeks	1.008 (0.999, 1.017)	**1.014 (1.001, 1.028)**
12 Weeks	1.006 (0.998, 1.014)	**1.015 (1.003, 1.026)**
13 Weeks	1.004 (0.996, 1.012)	**1.015 (1.004, 1.026)**
14 Weeks	1.002 (0.994, 1.009)	**1.015 (1.005, 1.026)**
15 Weeks	1.000 (0.992, 1.008)	**1.016 (1.004, 1.027)**
16 Weeks	0.998 (0.990, 1.006)	**1.016 (1.003, 1.029)**
17 Weeks	0.997 (0.988, 1.006)	**1.016 (1.002, 1.030)**
18 Weeks	0.996 (0.987, 1.005)	**1.016 (1.001, 1.032)**
19 Weeks	0.996 (0.987, 1.005)	**1.017 (1.002, 1.032)**
20 Weeks	0.997 (0.988, 1.006)	**1.017 (1.003, 1.031)**
21 Weeks	0.998 (0.990, 1.006)	**1.017 (1.004, 1.030)**
22 Weeks	1.000 (0.992, 1.008)	**1.017 (1.006, 1.029)**
23 Weeks	1.002 (0.995, 1.010)	**1.018 (1.006, 1.029)**
24 Weeks	1.005 (0.997, 1.013)	**1.018 (1.005, 1.031)**
25 Weeks	1.008 (0.999, 1.018)	**1.018 (1.002, 1.035)**
26 Weeks	**1.012 (1.000, 1.023)**	1.018 (0.998, 1.040)

27 Weeks	**1.015 (1.001, 1.029)**	1.019 (0.992, 1.046)
28 Weeks	**1.019 (1.002, 1.035)**	1.019 (0.987, 1.052)

Abbreviations: GDM, gestational diabetes mellitus; GDMA1, diet-controlled GDM; GDMA2, insulin-treated GDM; OR, odds ratio; 95% CI, 95% confidence interval

Note: Distributed lag non-linear models (DLNMs) incorporated with logistic regression was used to calculate adjusted ORs (95% CIs) each 10μg/m^3 increment in the concentrations of O_3 at a weekly level over the preconception period and pregnancy.

All models adjusted for maternal age, preconception BMI, educational level, occupation, gravidity, parity, conception season, gestational hypertension, newborn gender, and natural cubic splines with 3 degrees of freedom for ambient temperature and dew point.

Black bold indicate statistically significance (P <0.05) with positive effects.

Table S4. Modification effect of ambient temperature on the association of O_3 exposure with the risk of GDMA1 and GDMA2

Exposure	GDMA1 Adjusted OR (95% CI)				GDMA2 Adjusted OR (95% CI)			
	Low	Moderate	High	P for interaction	Low	Moderate	High	P for interaction
**Preconception**								
12 Weeks	0.663 (0.312, 1.409)	**1.111 (1.083, 1.138)**	1.452 (0.875, 2.412)	0.238	0.371 (0.179, 0.767)	0.972 (0.929, 1.016)	0.858 (0.444, 1.658)	0.033
11 Weeks	0.748 (0.399, 1.404)	**1.098 (1.075, 1.121)**	1.492 (0.977, 2.278)	0.179	0.438 (0.239, 0.804)	0.973 (0.937, 1.011)	0.870 (0.503, 1.504)	0.034
10 Weeks	0.843 (0.501, 1.418)	**1.085 (1.066, 1.104)**	**1.530 (1.078, 2.170)**	0.100	0.517 (0.313, 0.856)	0.975 (0.945, 1.006)	0.882 (0.563, 1.381)	0.044
9 Weeks	0.947 (0.614, 1.460)	**1.073 (1.057, 1.089)**	**1.564 (1.169, 2.094)**	0.034	0.610 (0.398, 0.934)	0.977 (0.952, 1.002)	0.894 (0.617, 1.294)	0.087
8 Weeks	1.060 (0.726, 1.547)	**1.061 (1.048, 1.075)**	**1.593 (1.234, 2.057)**	0.008	0.717 (0.487, 1.055)	0.979 (0.958, 0.999)	0.906 (0.657, 1.248)	0.258
7 Weeks	1.180 (0.824, 1.691)	**1.051 (1.039, 1.063)**	**1.613 (1.263, 2.061)**	0.002	0.840 (0.574, 1.230)	0.981 (0.964, 0.998)	0.917 (0.675, 1.245)	0.666
6 Weeks	1.305 (0.901, 1.890)	**1.042 (1.030, 1.053)**	**1.622 (1.259, 2.091)**	0.001	0.981 (0.657, 1.466)	0.983 (0.967, 0.998)	0.928 (0.675, 1.274)	0.939
5 Weeks	1.431 (0.964, 2.125)	**1.034 (1.022, 1.045)**	**1.618 (1.234, 2.122)**	0.001	1.141 (0.741, 1.755)	0.985 (0.970, 1.000)	0.938 (0.667, 1.320)	0.770
4 Weeks	**1.555 (1.023, 2.365)**	**1.027 (1.015, 1.039)**	**1.598 (1.198, 2.131)**	0.002	1.319 (0.836, 2.081)	0.987 (0.972, 1.003)	0.947 (0.658, 1.365)	0.451
3 Weeks	**1.672 (1.084, 2.576)**	**1.022 (1.010, 1.034)**	**1.560 (1.159, 2.101)**	0.002	1.516 (0.949, 2.421)	0.990 (0.974, 1.007)	0.956 (0.655, 1.395)	0.202
2 Weeks	**1.775 (1.155, 2.728)**	**1.019 (1.007, 1.031)**	**1.504 (1.119, 2.022)**	0.001	**1.730 (1.089, 2.748)**	0.993 (0.977, 1.009)	0.963 (0.661, 1.403)	0.063
1 Weeks	**1.861 (1.235, 2.804)**	**1.017 (1.006, 1.029)**	**1.432 (1.080, 1.900)**	0.001	**1.958 (1.261, 3.040)**	0.996 (0.981, 1.012)	0.970 (0.677, 1.388)	0.011
**After pregnancy**								
1 Weeks	**1.926 (1.316, 2.819)**	**1.017 (1.007, 1.028)**	**1.351 (1.038, 1.757)**	0.001	**2.194 (1.459, 3.302)**	1 (0.985, 1.014)	0.974 (0.699, 1.358)	0.001
2 Weeks	**1.967 (1.382, 2.800)**	**1.019 (1.009, 1.029)**	1.265 (0.990, 1.615)	<0.001	**2.43 (1.662, 3.552)**	1.003 (0.989, 1.016)	0.977 (0.72, 1.327)	<0.001
3 Weeks	**1.985 (1.417, 2.779)**	**1.021 (1.011, 1.031)**	1.180 (0.934, 1.491)	<0.001	**2.653 (1.841, 3.821)**	1.006 (0.993, 1.019)	0.979 (0.733, 1.307)	<0.001
4 Weeks	**1.977 (1.409, 2.776)**	**1.023 (1.014, 1.033)**	1.101 (0.870, 1.393)	0.001	**2.849 (1.965, 4.131)**	1.009 (0.996, 1.022)	0.978 (0.732, 1.306)	<0.001
5 Weeks	**1.946 (1.358, 2.788)**	**1.026 (1.016, 1.036)**	1.029 (0.802, 1.319)	0.002	**3.005 (2.02, 4.47)**	1.012 (0.998, 1.026)	0.975 (0.718, 1.324)	<0.001
6 Weeks	**1.892 (1.281, 2.793)**	**1.029 (1.018, 1.040)**	0.967 (0.739, 1.265)	0.008	**3.105 (2.016, 4.784)**	1.014 (0.999, 1.03)	0.969 (0.695, 1.352)	<0.001

7 Weeks	**1.817 (1.195, 2.762)**	**1.031 (1.019, 1.042)**	0.917 (0.687, 1.223)	0.022	**3.137 (1.97, 4.996)**	1.016 (0.999, 1.033)	0.962 (0.672, 1.377)	<0.001
8 Weeks	**1.725 (1.114, 2.670)**	**1.032 (1.020, 1.044)**	0.880 (0.651, 1.188)	0.041	**3.094 (1.903, 5.028)**	1.017 (0.999, 1.035)	0.951 (0.653, 1.385)	<0.001
9 Weeks	**1.618 (1.045, 2.504)**	**1.032 (1.020, 1.044)**	0.856 (0.634, 1.156)	0.062	**2.971 (1.827, 4.83)**	1.017 (0.999, 1.035)	0.938 (0.644, 1.368)	<0.001
10 Weeks	1.501 (0.987, 2.282)	**1.031 (1.019, 1.042)**	0.846 (0.634, 1.129)	0.086	**2.782 (1.746, 4.432)**	1.017 (0.999, 1.033)	0.923 (0.643, 1.326)	<0.001
11 Weeks	1.381 (0.934, 2.040)	**1.029 (1.018, 1.039)**	0.847 (0.648, 1.108)	0.123	**2.551 (1.655, 3.933)**	**1.015 (1.000, 1.031)**	0.907 (0.647, 1.27)	<0.001
12 Weeks	1.260 (0.879, 1.808)	**1.026 (1.016, 1.036)**	0.857 (0.668, 1.099)	0.197	**2.302 (1.547, 3.426)**	**1.014 (1.001, 1.028)**	0.89 (0.652, 1.215)	<0.001
13 Weeks	1.144 (0.814, 1.610)	**1.022 (1.013, 1.031)**	0.875 (0.691, 1.107)	0.350	**2.053 (1.416, 2.976)**	**1.012 (1.000, 1.025)**	0.874 (0.652, 1.171)	0.001
14 Weeks	1.035 (0.737, 1.453)	**1.018 (1.009, 1.027)**	0.898 (0.710, 1.136)	0.576	**1.817 (1.263, 2.615)**	1.011 (0.999, 1.023)	0.859 (0.642, 1.148)	0.004
15 Weeks	0.934 (0.654, 1.334)	**1.014 (1.005, 1.023)**	0.926 (0.724, 1.184)	0.696	**1.605 (1.101, 2.34)**	1.009 (0.997, 1.022)	0.845 (0.624, 1.145)	0.028
16 Weeks	0.843 (0.574, 1.239)	**1.010 (1.000, 1.020)**	0.956 (0.734, 1.246)	0.604	1.42 (0.947, 2.127)	1.008 (0.995, 1.022)	0.835 (0.602, 1.158)	0.133
17 Weeks	0.762 (0.504, 1.152)	1.006 (0.996, 1.017)	0.987 (0.743, 1.312)	0.417	1.264 (0.818, 1.952)	1.008 (0.993, 1.023)	0.827 (0.582, 1.176)	0.324
18 Weeks	0.691 (0.448, 1.065)	1.003 (0.992, 1.014)	1.016 (0.755, 1.369)	0.240	1.137 (0.72, 1.795)	1.008 (0.992, 1.024)	0.824 (0.57, 1.19)	0.491
19 Weeks	0.630 (0.408, 0.973)	1.001 (0.990, 1.012)	1.042 (0.772, 1.405)	0.110	1.039 (0.655, 1.648)	1.009 (0.993, 1.025)	0.825 (0.57, 1.194)	0.561
20 Weeks	0.577 (0.379, 0.878)	0.999 (0.988, 1.010)	1.062 (0.795, 1.419)	0.035	0.964 (0.615, 1.509)	1.011 (0.996, 1.027)	0.83 (0.581, 1.187)	0.546
21 Weeks	0.531 (0.358, 0.787)	0.998 (0.987, 1.008)	1.078 (0.821, 1.416)	0.006	0.906 (0.594, 1.383)	**1.014 (1, 1.029)**	0.84 (0.6, 1.175)	0.477
22 Weeks	0.490 (0.339, 0.708)	0.997 (0.987, 1.007)	1.090 (0.845, 1.406)	0.001	0.862 (0.581, 1.279)	**1.018 (1.005, 1.031)**	0.853 (0.624, 1.167)	0.386
23 Weeks	0.454 (0.319, 0.647)	0.997 (0.987, 1.007)	1.098 (0.859, 1.404)	<0.001	0.829 (0.57, 1.206)	**1.022 (1.009, 1.035)**	0.869 (0.643, 1.176)	0.317
24 Weeks	0.422 (0.292, 0.611)	0.997 (0.987, 1.008)	1.104 (0.855, 1.426)	<0.001	0.803 (0.548, 1.178)	**1.027 (1.012, 1.042)**	0.889 (0.648, 1.22)	0.305
25 Weeks	0.393 (0.258, 0.601)	0.998 (0.986, 1.010)	1.107 (0.827, 1.482)	<0.001	0.784 (0.512, 1.201)	**1.032 (1.014, 1.05)**	0.911 (0.633, 1.31)	0.361
26 Weeks	0.367 (0.220, 0.611)	0.999 (0.985, 1.012)	1.108 (0.781, 1.572)	0.001	0.769 (0.464, 1.276)	**1.038 (1.015, 1.061)**	0.935 (0.603, 1.451)	0.460
27 Weeks	0.343 (0.184, 0.637)	1.000 (0.984, 1.016)	1.108 (0.725, 1.693)	0.003	0.757 (0.411, 1.396)	**1.043 (1.015, 1.072)**	0.961 (0.564, 1.64)	0.565
28 Weeks	0.321 (0.152, 0.675)	1.001 (0.982, 1.020)	1.107 (0.666, 1.840)	0.010	0.746 (0.358, 1.555)	**1.049 (1.015, 1.085)**	0.989 (0.521, 1.878)	0.651

Abbreviations: GDM, gestational diabetes mellitus; GDMA1, diet-controlled GDM; GDMA2, insulin-treated GDM; OR, odds ratio; 95% CI, 95% confidence interval

Note: Weekly average temperature was divided into three levels using the 10th and 90th percentiles.

Distributed lag non-linear models (DLNMs) incorporated with logistic regression was used to calculate adjusted ORs (95% CIs) each 10μg/m^3 increment in the concentrations of O_3 at a weekly level over the preconception period and pregnancy.
All models adjusted for maternal age, preconception BMI, educational level, occupation, gravidity, parity, conception season, gestational hypertension, newborn gender, and natural cubic splines with 3 degrees of freedom for dew point.
Black bold indicate statistically significance ($P < 0.05$) with positive effects.

Table S5. Subgroup analysis for the association of O_3 exposure with the risk of GDMA1 and GDMA2

Exposure	GDMA1			GDMA2	
	Case/N	Adjusted OR (95% CI) Preconception (12 week)	Adjusted OR (95% CI) After pregnancy (28 week)	Case/N	Adjusted OR (95% CI) After pregnancy (25 week)
**Maternal age**					
<35 years	1364/8503	**1.086 (1.055, 1.118)**	**1.041 (1.021, 1.061)**	201/8503	0.977 (0.957, 0.997)
≥35 years	620/2988	1.026 (0.983, 1.071)	0.973 (0.945, 1.001)	138/2988	0.991 (0.966, 1.017)
P for interaction		0.001	0.019		0.004
**Preconception overweight**					
Yes	1093/8007	**1.090 (1.050, 1.131)**	**1.054 (1.029, 1.080)**	232/8007	0.989 (0.970, 1.008)
No	891/3484	**1.050 (1.017, 1.084)**	0.995 (0.974, 1.017)	107/3484	0.985 (0.957, 1.014)
P for interaction		< 0.001	< 0.001		< 0.001
**Gravidity**					
Nulligravida	866/5377	**1.123 (1.082, 1.165)**	**1.046 (1.019, 1.073)**	128/5377	1.009 (0.980, 1.038)
Multigravida	1118/6114	1.020 (0.988, 1.053)	0.997 (0.977, 1.019)	211/6114	0.970 (0.951, 0.99)
P for interaction		0.439	0.163		0.286
**Parity**					
Nullipara	1330/7808	**1.077 (1.046, 1.110)**	**1.024 (1.003, 1.045)**	7808	0.999 (0.978, 1.020)
Multipara	654/3683	1.039 (0.997, 1.083)	1.027 (0.999, 1.054)	3683	0.964 (0.940, 0.989)
P for interaction		0.010	0.051		0.561
**Gestational hypertension**					
Yes	231/914	**1.209 (1.121, 1.305)**	**1.071 (1.019, 1.126)**	48/914	1.036 (0.993, 1.081)
No	1753 /10,577	**1.047 (1.021, 1.074)**	**1.018 (1.001, 1.036)**	291 /10,577	0.993 (0.976, 1.011)

P for interaction		0.007	0.049		0.342
**Family history of diabetes**					
Yes	269/931	1.017 (0.947, 1.092)	**1.091 (1.042, 1.142)**	65/931	0.933 (0.901, 0.968)
No	1715/10,560	**1.055 (1.028, 1.083)**	1.010 (0.992, 1.027)	274/10,560	**1.031 (1.012, 1.049)**
P for interaction		< 0.001	< 0.001		< 0.001

Abbreviations: GDM, gestational diabetes mellitus; GDMA1, diet-controlled GDM; GDMA2, insulin-treated GDM; OR, odds ratio; 95% CI, 95% confidence interval

Note: Results at the sensitive (with the highest effect estimates) exposure weeks of O_3 exposure for GDMA1 (preconception 12th week and 28th week after pregnancy) and GDMA2 (25th week after pregnancy) were selected for result presentation

All models adjusted for maternal age, preconception BMI, educational level, occupation, gravidity, parity, conception season, gestational hypertension, newborn gender, and natural cubic splines with 3 degrees of freedom for ambient temperature and dew point (except for the stratification variables).

Black bold indicate statistically significance (P <0.05) with positive effects.

Table S6. Sensitivity analysis of the association of O_3 exposure with the risk of GDMA1 and GDMA2 using the Class I concentration limits (100 μg/m^3) of GB3095-2012 as the reference in DLNM models

Exposure	GDMA1<br>Adjusted OR (95% CI)	GDMA2<br>Adjusted OR (95% CI)
**Preconception**		
12 Weeks	**1.033 (1.009, 1.059)**	0.993 (0.952, 1.036)
11 Weeks	**1.029 (1.007, 1.051)**	0.995 (0.960, 1.031)
10 Weeks	**1.024 (1.006, 1.043)**	0.997 (0.968, 1.027)
9 Weeks	**1.020 (1.004, 1.035)**	0.999 (0.975, 1.024)
8 Weeks	**1.016 (1.003, 1.029)**	1.001 (0.981, 1.021)
7 Weeks	**1.012 (1.000, 1.023)**	1.003 (0.987, 1.019)
6 Weeks	1.008 (0.998, 1.018)	1.004 (0.991, 1.019)
5 Weeks	1.005 (0.995, 1.015)	1.006 (0.993, 1.019)
4 Weeks	1.002 (0.992, 1.012)	1.007 (0.994, 1.021)
3 Weeks	1.000 (0.990, 1.010)	1.009 (0.995, 1.022)
2 Weeks	0.998 (0.988, 1.009)	1.009 (0.996, 1.023)
1 Weeks	0.997 (0.987, 1.008)	1.010 (0.997, 1.024)
**After pregnancy**		
1 Weeks	0.997 (0.987, 1.007)	1.011 (0.998, 1.024)
2 Weeks	0.997 (0.987, 1.006)	1.011 (0.999, 1.023)
3 Weeks	0.997 (0.987, 1.006)	1.011 (1.000, 1.023)
4 Weeks	0.997 (0.988, 1.007)	**1.012 (1.000, 1.023)**
5 Weeks	0.998 (0.988, 1.008)	1.012 (1.000, 1.024)
6 Weeks	0.998 (0.988, 1.009)	1.012 (0.999, 1.025)
7 Weeks	0.999 (0.988, 1.009)	1.013 (0.999, 1.027)
8 Weeks	0.999 (0.988, 1.010)	1.013 (0.999, 1.028)
9 Weeks	0.999 (0.988, 1.010)	1.014 (0.999, 1.028)
10 Weeks	0.999 (0.988, 1.009)	**1.015 (1.001, 1.029)**
11 Weeks	0.998 (0.988, 1.008)	**1.016 (1.002, 1.029)**
12 Weeks	0.998 (0.988, 1.007)	**1.017 (1.004, 1.029)**
13 Weeks	0.997 (0.987, 1.006)	**1.018 (1.006, 1.029)**
14 Weeks	0.996 (0.987, 1.005)	**1.019 (1.007, 1.030)**
15 Weeks	0.995 (0.986, 1.005)	**1.020 (1.008, 1.031)**
16 Weeks	0.994 (0.985, 1.004)	**1.020 (1.008, 1.033)**
17 Weeks	0.994 (0.984, 1.004)	**1.021 (1.008, 1.035)**
18 Weeks	0.994 (0.984, 1.004)	**1.022 (1.008, 1.036)**
19 Weeks	0.994 (0.984, 1.004)	**1.022 (1.008, 1.036)**
20 Weeks	0.994 (0.984, 1.004)	**1.022 (1.008, 1.035)**
21 Weeks	0.995 (0.985, 1.004)	**1.021 (1.009, 1.034)**
22 Weeks	0.995 (0.986, 1.005)	**1.021 (1.009, 1.033)**
23 Weeks	0.996 (0.987, 1.006)	**1.020 (1.008, 1.032)**
24 Weeks	0.998 (0.988, 1.007)	**1.019 (1.006, 1.033)**
25 Weeks	0.999 (0.988, 1.010)	**1.018 (1.002, 1.034)**

26 Weeks	1.000 (0.988, 1.013)	1.017 (0.998, 1.037)
27 Weeks	1.002 (0.988, 1.016)	1.016 (0.993, 1.040)
28 Weeks	1.003 (0.987, 1.020)	1.015 (0.987, 1.043)

Abbreviations: GDM, gestational diabetes mellitus; GDMA1, diet-controlled GDM; GDMA2, insulin-treated GDM; OR, odds ratio; 95% CI, 95% confidence interval

Note: Distributed lag non-linear models (DLNMs) incorporated with logistic regression was used to calculate adjusted ORs (95% CIs) each 10μg/m^3 increment in the concentrations of O_3 at a weekly level over the preconception period and pregnancy.

All models adjusted for maternal age, preconception BMI, educational level, occupation, gravidity, parity, conception season, gestational hypertension, newborn gender, and natural cubic splines with 3 degrees of freedom for ambient temperature and dew point.

Black bold indicate statistically significance (P <0.05) with positive effects.

Table S7. Sensitivity analysis of the association of O_3 exposure with the risk of GDMA1 and GDMA2, with further adjustments of maternal smoking and drinking covariates

Exposure	GDMA1<br>Adjusted OR (95% CI)	GDMA2<br>Adjusted OR (95% CI)
**Preconception**		
12 Weeks	**1.062 (1.036, 1.088)**	0.994 (0.947, 1.044)
11 Weeks	**1.054 (1.032, 1.075)**	0.996 (0.956, 1.037)
10 Weeks	**1.046 (1.028, 1.064)**	0.998 (0.965, 1.032)
9 Weeks	**1.038 (1.023, 1.052)**	0.999 (0.973, 1.026)
8 Weeks	**1.031 (1.019, 1.042)**	1.001 (0.980, 1.022)
7 Weeks	**1.024 (1.014, 1.034)**	1.002 (0.985, 1.019)
6 Weeks	**1.018 (1.009, 1.027)**	1.004 (0.989, 1.018)
5 Weeks	**1.012 (1.004, 1.021)**	1.005 (0.991, 1.019)
4 Weeks	1.008 (0.999, 1.017)	1.006 (0.992, 1.021)
3 Weeks	1.005 (0.996, 1.014)	1.007 (0.992, 1.022)
2 Weeks	1.003 (0.993, 1.012)	1.008 (0.993, 1.024)
1 Weeks	1.002 (0.993, 1.011)	1.009 (0.995, 1.024)
**After pregnancy**		
1 Weeks	1.001 (0.993, 1.010)	1.010 (0.997, 1.023)
2 Weeks	1.002 (0.994, 1.010)	1.010 (0.998, 1.022)
3 Weeks	1.003 (0.995, 1.011)	1.011 (0.999, 1.022)
4 Weeks	1.005 (0.997, 1.013)	**1.011 (1.000, 1.022)**
5 Weeks	1.006 (0.998, 1.015)	1.011 (0.999, 1.023)
6 Weeks	1.008 (0.999, 1.017)	1.012 (0.998, 1.025)
7 Weeks	1.009 (0.999 1.019)	1.012 (0.997, 1.027)
8 Weeks	**1.010 (1.000, 1.020)**	1.012 (0.996, 1.028)
9 Weeks	**1.010 (1.000, 1.020)**	1.013 (0.997, 1.029)
10 Weeks	1.009 (0.999, 1.018)	1.013 (0.998, 1.028)
11 Weeks	1.008 (0.999, 1.017)	1.013 (0.999, 1.027)
12 Weeks	1.006 (0.998, 1.014)	**1.014 (1.002, 1.026)**
13 Weeks	1.004 (0.996, 1.012)	**1.014 (1.004, 1.025)**
14 Weeks	1.002 (0.994, 1.009)	**1.015 (1.004, 1.025)**
15 Weeks	1.000 (0.992, 1.008)	**1.015 (1.004, 1.027)**
16 Weeks	0.998 (0.990, 1.006)	**1.016 (1.003, 1.028)**
17 Weeks	0.997 (0.988, 1.006)	**1.016 (1.002, 1.030)**
18 Weeks	0.996 (0.987, 1.005)	**1.016 (1.001, 1.032)**
19 Weeks	0.996 (0.987, 1.005)	**1.017 (1.001, 1.032)**
20 Weeks	0.997 (0.988, 1.005)	**1.017 (1.003, 1.031)**
21 Weeks	0.998 (0.990, 1.006)	**1.017 (1.004, 1.030)**
22 Weeks	1.000 (0.992, 1.008)	**1.017 (1.006, 1.029)**
23 Weeks	1.002 (0.995, 1.010)	**1.017 (1.006, 1.029)**
24 Weeks	1.005 (0.997, 1.013)	**1.018 (1.005, 1.031)**
25 Weeks	1.008 (0.999, 1.018)	**1.018 (1.001, 1.034)**

26 Weeks	**1.012 (1.000, 1.023)**	1.018 (0.997, 1.039)
27 Weeks	**1.015 (1.002, 1.029)**	1.018 (0.992, 1.045)
28 Weeks	**1.019 (1.003, 1.035)**	1.018 (0.986, 1.051)

Abbreviations: GDM, gestational diabetes mellitus; GDMA1, diet-controlled GDM; GDMA2, insulin-treated GDM; OR, odds ratio; 95% CI, 95% confidence interval

Note: Distributed lag non-linear models (DLNMs) incorporated with logistic regression was used to calculate adjusted ORs (95% CIs) each 10μg/m^3 increment in the concentrations of O_3 at a weekly level over the preconception period and pregnancy.

All models adjusted for maternal age, preconception BMI, educational level, occupation, gravidity, parity, maternal smoking, maternal drinking, conception season, gestational hypertension, newborn gender, and natural cubic splines with 3 degrees of freedom for ambient temperature and dew point.

Black bold indicate statistically significance (P <0.05) with positive effects.

Table S8. Sensitivity analysis of the association of O_3 exposure with the risk of GDMA1 and GDMA2 in two-pollutant models

Exposure	GDMA1<br>Adjusted OR (95% CI)	GDMA2<br>Adjusted OR (95% CI)
**Preconception**		
12 Weeks	**1.098 (1.071, 1.125)**	0.994 (0.946, 1.044)
11 Weeks	**1.085 (1.062, 1.108)**	0.995 (0.955, 1.038)
10 Weeks	**1.073 (1.054, 1.092)**	0.997 (0.963, 1.032)
9 Weeks	**1.061 (1.046, 1.076)**	0.999 (0.971, 1.026)
8 Weeks	**1.050 (1.037, 1.062)**	1.000 (0.979, 1.022)
7 Weeks	**1.039 (1.029, 1.050)**	1.002 (0.984, 1.019)
6 Weeks	**1.030 (1.021, 1.039)**	1.003 (0.988, 1.018)
5 Weeks	**1.022 (1.013, 1.031)**	1.004 (0.990, 1.019)
4 Weeks	**1.015 (1.006, 1.024)**	1.006 (0.991, 1.020)
3 Weeks	**1.010 (1.001, 1.019)**	1.007 (0.992, 1.022)
2 Weeks	1.006 (0.997, 1.016)	1.008 (0.993, 1.023)
1 Weeks	1.004 (0.995, 1.014)	1.009 (0.994, 1.023)
**After pregnancy**		
1 Weeks	1.004 (0.995, 1.013)	1.009 (0.996, 1.023)
2 Weeks	1.004 (0.996, 1.013)	1.010 (0.998, 1.022)
3 Weeks	1.006 (0.998, 1.014)	1.011 (0.999, 1.021)
4 Weeks	1.008 (0.999, 1.016)	**1.011 (1.000, 1.022)**
5 Weeks	**1.010 (1.002, 1.018)**	1.011 (0.999, 1.023)
6 Weeks	**1.012 (1.003, 1.021)**	1.012 (0.998, 1.025)
7 Weeks	**1.014 (1.004, 1.023)**	1.012 (0.997, 1.027)
8 Weeks	**1.015 (1.005, 1.025)**	1.013 (0.997, 1.029)
9 Weeks	**1.015 (1.005, 1.025)**	1.013 (0.997, 1.029)
10 Weeks	**1.014 (1.005, 1.024)**	1.013 (0.998, 1.029)
11 Weeks	**1.013 (1.004, 1.021)**	**1.014 (1.000, 1.027)**
12 Weeks	**1.010 (1.002, 1.019)**	**1.014 (1.002, 1.026)**
13 Weeks	**1.008 (1.000, 1.016)**	**1.015 (1.004, 1.025)**
14 Weeks	1.005 (0.998, 1.013)	**1.015 (1.005, 1.025)**
15 Weeks	1.003 (0.995, 1.011)	**1.015 (1.004, 1.027)**
16 Weeks	1.001 (0.993, 1.009)	**1.016 (1.003, 1.029)**
17 Weeks	0.999 (0.990, 1.008)	**1.016 (1.002, 1.030)**
18 Weeks	0.998 (0.989, 1.007)	**1.016 (1.001, 1.032)**
19 Weeks	0.998 (0.989, 1.007)	**1.017 (1.002, 1.032)**
20 Weeks	0.999 (0.99, 1.007)	**1.017 (1.003, 1.032)**
21 Weeks	1.000 (0.992, 1.008)	**1.017 (1.004, 1.030)**
22 Weeks	1.002 (0.994, 1.010)	**1.017 (1.006, 1.029)**
23 Weeks	1.005 (0.997, 1.013)	**1.018 (1.006, 1.029)**
24 Weeks	**1.008 (1.000, 1.017)**	**1.018 (1.005, 1.031)**
25 Weeks	**1.012 (1.003, 1.022)**	**1.018 (1.002, 1.034)**
26 Weeks	**1.016 (1.005, 1.027)**	1.018 (0.997, 1.039)

27 Weeks	**1.020 (1.006, 1.034)**	1.018 (0.992, 1.045)
28 Weeks	**1.025 (1.008, 1.041)**	1.018 (0.986, 1.051)

Abbreviations: GDM, gestational diabetes mellitus; GDMA1, diet-controlled GDM; GDMA2, insulin-treated GDM; OR, odds ratio; 95% CI, 95% confidence interval

Note: Distributed lag non-linear models (DLNMs) incorporated with logistic regression was used to calculate adjusted ORs (95% CIs) each 10μg/m^3 increment in the concentrations of O_3 at a weekly level over the preconception period and pregnancy.

All models adjusted for maternal age, preconception BMI, educational level, occupation, gravidity, parity, conception season, gestational hypertension, newborn gender, $PM_{2.5}$ concentration and natural cubic splines with 3 degrees of freedom for ambient temperature and dew point.

Black bold indicate statistically significance (P <0.05) with positive effects.

Table S9. Sensitivity analysis of modification effect of ambient temperature (stratified by the 25th and 75th percentiles) on the association of O_3 exposure with the risk of GDMA1 and GDMA2

Exposure	GDMA1 Adjusted OR (95% CI)				GDMA2 Adjusted OR (95% CI)			
	Low	Moderate	High	P for interaction	Low	Moderate	High	P for interaction
**Preconception**								
12 Weeks	0.558 (0.398, 0.783)	**1.057 (1.016, 1.099)**	1.278 (0.909, 1.798)	0.001	0.798 (0.618, 1.030)	0.945 (0.902, 0.991)	0.787 (0.528, 1.173)	0.303
11 Weeks	0.616 (0.464, 0.819)	**1.057 (1.021, 1.094)**	1.224 (0.918, 1.633)	0.001	0.831 (0.669, 1.033)	0.950 (0.912, 0.990)	0.752 (0.538, 1.051)	0.203
10 Weeks	0.679 (0.535, 0.862)	**1.057 (1.025, 1.090)**	1.174 (0.921, 1.497)	0.001	0.866 (0.720, 1.042)	0.956 (0.922, 0.990)	0.719 (0.544, 0.951)	0.088
9 Weeks	0.746 (0.609, 0.914)	**1.057 (1.028, 1.086)**	1.129 (0.916, 1.392)	0.003	0.900 (0.766, 1.059)	0.961 (0.931, 0.991)	0.689 (0.543, 0.875)	0.020
8 Weeks	0.816 (0.680, 0.980)	**1.056 (1.029, 1.084)**	1.090 (0.900, 1.320)	0.022	0.934 (0.805, 1.085)	0.966 (0.939, 0.994)	0.664 (0.535, 0.823)	0.003
7 Weeks	0.887 (0.742, 1.059)	**1.056 (1.029, 1.083)**	1.058 (0.876, 1.277)	0.163	0.967 (0.835, 1.121)	0.971 (0.946, 0.998)	0.642 (0.520, 0.792)	0.001
6 Weeks	0.956 (0.795, 1.149)	**1.055 (1.028, 1.083)**	1.033 (0.848, 1.258)	0.573	0.998 (0.856, 1.163)	0.977 (0.951, 1.003)	0.625 (0.502, 0.779)	<0.001
5 Weeks	1.020 (0.839, 1.241)	**1.054 (1.026, 1.082)**	1.017 (0.825, 1.253)	0.899	1.025 (0.873, 1.205)	0.982 (0.956, 1.009)	0.613 (0.485, 0.775)	<0.001
4 Weeks	1.077 (0.876, 1.324)	**1.053 (1.024, 1.082)**	1.010 (0.810, 1.258)	0.913	1.049 (0.886, 1.240)	0.988 (0.961, 1.015)	0.607 (0.474, 0.777)	<0.001
3 Weeks	1.122 (0.907, 1.387)	**1.051 (1.022, 1.080)**	1.014 (0.809, 1.270)	0.793	1.067 (0.899, 1.265)	0.994 (0.966, 1.022)	0.607 (0.471, 0.782)	0.001
2 Weeks	1.152 (0.934, 1.422)	**1.048 (1.020, 1.078)**	1.029 (0.823, 1.287)	0.674	1.078 (0.911, 1.276)	1.000 (0.972, 1.028)	0.614 (0.478, 0.789)	<0.001
1 Weeks	1.166 (0.953, 1.425)	**1.046 (1.018, 1.074)**	1.057 (0.854, 1.310)	0.574	1.084 (0.923, 1.272)	1.006 (0.978, 1.034)	0.628 (0.495, 0.798)	<0.001
**After pregnancy**								
1 Weeks	1.165 (0.965, 1.405)	**1.043 (1.016, 1.070)**	1.096 (0.897, 1.340)	0.466	1.083 (0.932, 1.258)	1.012 (0.985, 1.040)	0.65 (0.521, 0.811)	<0.001
2 Weeks	1.152 (0.967, 1.372)	**1.039 (1.014, 1.065)**	1.144 (0.948, 1.381)	0.327	1.077 (0.936, 1.239)	1.018 (0.992, 1.045)	0.678 (0.552, 0.833)	<0.001
3 Weeks	1.130 (0.955, 1.337)	**1.036 (1.011, 1.061)**	**1.198 (1.000, 1.436)**	0.184	1.067 (0.932, 1.222)	1.024 (0.998, 1.050)	0.712 (0.584, 0.867)	<0.001
4 Weeks	1.103 (0.931, 1.307)	**1.032 (1.008, 1.058)**	**1.256 (1.047, 1.506)**	0.087	1.055 (0.919, 1.210)	**1.029 (1.004, 1.056)**	0.751 (0.615, 0.917)	0.008
5 Weeks	1.073 (0.898, 1.283)	**1.029 (1.004, 1.055)**	**1.313 (1.085, 1.590)**	0.042	1.040 (0.899, 1.203)	**1.035 (1.009, 1.061)**	0.795 (0.643, 0.982)	0.053

6 Weeks	1.044 (0.861, 1.265)	**1.026 (1.001, 1.052)**	**1.367 (1.115, 1.678)**	0.024	1.024 (0.875, 1.198)	**1.039 (1.014, 1.066)**	0.843 (0.670, 1.059)	0.201
7 Weeks	1.017 (0.828, 1.249)	1.023 (0.997, 1.050)	**1.413 (1.137, 1.756)**	0.015	1.009 (0.853, 1.193)	**1.043 (1.017, 1.070)**	0.893 (0.698, 1.142)	0.435
8 Weeks	0.995 (0.804, 1.232)	1.021 (0.994, 1.048)	**1.445 (1.153, 1.811)**	0.011	0.994 (0.834, 1.184)	**1.047 (1.020, 1.074)**	0.944 (0.731, 1.220)	0.626
9 Weeks	0.980 (0.792, 1.214)	1.019 (0.992, 1.046)	**1.459 (1.164, 1.828)**	0.008	0.980 (0.822, 1.168)	**1.049 (1.023, 1.076)**	0.994 (0.769, 1.285)	0.697
10 Weeks	0.973 (0.792, 1.195)	1.018 (0.992, 1.044)	**1.453 (1.169, 1.806)**	0.006	0.969 (0.818, 1.148)	**1.051 (1.025, 1.077)**	1.042 (0.814, 1.333)	0.651
11 Weeks	0.972 (0.802, 1.179)	1.017 (0.992, 1.043)	**1.431 (1.166, 1.756)**	0.005	0.960 (0.819, 1.126)	**1.051 (1.026, 1.077)**	1.085 (0.862, 1.366)	0.523
12 Weeks	0.977 (0.817, 1.169)	1.017 (0.993, 1.042)	**1.396 (1.152, 1.691)**	0.005	0.954 (0.823, 1.107)	**1.051 (1.026, 1.077)**	1.123 (0.908, 1.388)	0.370
13 Weeks	0.987 (0.833, 1.17)	1.017 (0.993, 1.041)	**1.352 (1.126, 1.624)**	0.010	0.952 (0.828, 1.094)	**1.05 (1.025, 1.076)**	1.153 (0.944, 1.408)	0.253
14 Weeks	1.002 (0.847, 1.185)	1.017 (0.994, 1.042)	**1.303 (1.086, 1.563)**	0.030	0.952 (0.831, 1.091)	**1.048 (1.023, 1.074)**	1.174 (0.965, 1.429)	0.202
15 Weeks	1.020 (0.856, 1.215)	1.018 (0.994, 1.044)	**1.251 (1.036, 1.512)**	0.106	0.957 (0.833, 1.099)	**1.046 (1.020, 1.072)**	1.186 (0.968, 1.453)	0.219
16 Weeks	1.040 (0.863, 1.255)	1.019 (0.994, 1.046)	1.201 (0.982, 1.469)	0.282	0.966 (0.835, 1.118)	**1.042 (1.016, 1.069)**	1.186 (0.954, 1.474)	0.303
17 Weeks	1.064 (0.870, 1.300)	1.021 (0.994, 1.048)	1.153 (0.931, 1.429)	0.504	0.980 (0.839, 1.144)	**1.038 (1.012, 1.066)**	1.173 (0.929, 1.482)	0.452
18 Weeks	1.088 (0.882, 1.342)	1.022 (0.995, 1.051)	1.111 (0.888, 1.390)	0.655	0.999 (0.849, 1.176)	**1.033 (1.007, 1.061)**	1.148 (0.899, 1.467)	0.646
19 Weeks	1.113 (0.901, 1.376)	1.024 (0.996, 1.053)	1.076 (0.859, 1.349)	0.683	1.025 (0.868, 1.209)	**1.028 (1.001, 1.055)**	1.110 (0.867, 1.423)	0.831
20 Weeks	1.139 (0.927, 1.400)	1.026 (0.998, 1.054)	1.047 (0.840, 1.306)	0.605	1.056 (0.897, 1.244)	1.022 (0.996, 1.049)	1.062 (0.834, 1.353)	0.884
21 Weeks	1.165 (0.959, 1.416)	**1.028 (1.001, 1.055)**	1.024 (0.831, 1.263)	0.458	1.095 (0.935, 1.282)	1.015 (0.990, 1.041)	1.006 (0.799, 1.266)	0.652
22 Weeks	1.192 (0.992, 1.432)	**1.030 (1.004, 1.056)**	1.006 (0.826, 1.225)	0.294	1.139 (0.978, 1.326)	1.008 (0.984, 1.034)	0.944 (0.761, 1.172)	0.250
23 Weeks	**1.219 (1.021, 1.455)**	**1.032 (1.007, 1.058)**	0.991 (0.820, 1.198)	0.171	**1.189 (1.024, 1.381)**	1.001 (0.977, 1.026)	0.88 (0.715, 1.083)	0.038
24 Weeks	**1.246 (1.039, 1.495)**	**1.034 (1.009, 1.060)**	0.979 (0.808, 1.187)	0.116	**1.245 (1.067, 1.453)**	0.993 (0.969, 1.019)	0.815 (0.659, 1.008)	0.003
25 Weeks	**1.274 (1.041, 1.560)**	**1.036 (1.009, 1.064)**	0.970 (0.786, 1.197)	0.114	**1.307 (1.104, 1.547)**	0.986 (0.959, 1.013)	0.751 (0.593, 0.951)	<0.001
26 Weeks	**1.303 (1.027, 1.653)**	**1.039 (1.009, 1.069)**	0.963 (0.754, 1.229)	0.148	**1.374 (1.133, 1.667)**	0.978 (0.949, 1.007)	0.689 (0.523, 0.909)	<0.001
27 Weeks	**1.332 (1.002, 1.771)**	**1.041 (1.007, 1.076)**	0.956 (0.716, 1.277)	0.203	**1.447 (1.155, 1.813)**	0.97 (0.938, 1.003)	0.631 (0.454, 0.879)	<0.001
28 Weeks	1.362 (0.970, 1.911)	**1.043 (1.004, 1.084)**	0.951 (0.675, 1.339)	0.267	**1.525 (1.172, 1.983)**	0.962 (0.926, 0.999)	0.578 (0.390, 0.856)	<0.001

Abbreviations: GDM, gestational diabetes mellitus; GDMA1, diet-controlled GDM; GDMA2, insulin-treated GDM; OR, odds ratio; 95% CI, 95% confidence interval

Note: Weekly average temperature was divided into three levels using the 25th and 75th percentiles.

Distributed lag non-linear models (DLNMs) incorporated with logistic regression was used to calculate adjusted ORs (95% CIs) each 10μg/m^3 increment in the concentrations of O_3 at a weekly level over the preconception period and pregnancy.
All models adjusted for maternal age, preconception BMI, educational level, occupation, gravidity, parity, conception season, gestational hypertension, newborn gender, and natural cubic splines with 3 degrees of freedom for dew point.
Black bold indicate statistically significance ($P < 0.05$) with positive effects.

Table S10. Sensitivity analysis of modification effect of ambient temperature (stratified by the 5th and 95th percentiles) on the association of O_3 exposure with the risk of GDMA1 and GDMA2

Exposure	GDMA1 Adjusted OR (95% CI)				GDMA2 Adjusted OR (95% CI)			
	Low	Moderate	High	P for interaction	Low	Moderate	High	P for interaction
**Preconception**								
12 Weeks	1.129 (0.323, 3.943)	**1.085 (1.060, 1.110)**	0.865 (0.356, 2.100)	0.881	0.164 (0.055, 0.492)	1.004 (0.961, 1.048)	0.450 (0.160, 1.268)	0.002
11 Weeks	1.096 (0.386, 3.109)	**1.074 (1.053, 1.095)**	1.083 (0.514, 2.282)	0.999	0.260 (0.104, 0.647)	1.002 (0.966, 1.040)	0.458 (0.193, 1.084)	0.003
10 Weeks	1.066 (0.452, 2.514)	**1.063 (1.046, 1.080)**	1.349 (0.726, 2.506)	0.754	0.408 (0.192, 0.867)	1.001 (0.971, 1.031)	0.466 (0.230, 0.945)	0.007
9 Weeks	1.040 (0.511, 2.119)	**1.053 (1.039, 1.067)**	1.660 (0.987, 2.793)	0.229	0.633 (0.337, 1.189)	0.999 (0.975, 1.024)	0.474 (0.264, 0.850)	0.016
8 Weeks	1.021 (0.550, 1.894)	**1.043 (1.032, 1.055)**	**2.011 (1.274, 3.174)**	0.019	0.960 (0.548, 1.685)	0.998 (0.978, 1.018)	0.482 (0.290, 0.800)	0.019
7 Weeks	1.008 (0.560, 1.814)	**1.034 (1.024, 1.044)**	**2.383 (1.545, 3.674)**	0.001	1.417 (0.818, 2.455)	0.997 (0.981, 1.013)	0.490 (0.302, 0.793)	0.007
6 Weeks	1.005 (0.548, 1.843)	**1.026 (1.016, 1.035)**	**2.746 (1.765, 4.275)**	<0.001	**2.019 (1.133, 3.599)**	0.996 (0.982, 1.011)	0.497 (0.301, 0.821)	0.001
5 Weeks	1.012 (0.529, 1.938)	**1.018 (1.009, 1.028)**	**3.063 (1.917, 4.893)**	<0.001	**2.759 (1.480, 5.144)**	0.996 (0.982, 1.011)	0.505 (0.294, 0.865)	<0.001
4 Weeks	1.032 (0.517, 2.061)	**1.012 (1.003, 1.022)**	**3.287 (2.003, 5.392)**	<0.001	**3.589 (1.849, 6.970)**	0.996 (0.982, 1.011)	0.512 (0.288, 0.910)	<0.001
3 Weeks	1.067 (0.521, 2.185)	1.007 (0.997, 1.017)	**3.375 (2.025, 5.626)**	<0.001	**4.415 (2.224, 8.765)**	0.997 (0.982, 1.012)	0.518 (0.286, 0.941)	<0.001
2 Weeks	1.12 (0.548, 2.289)	1.004 (0.994, 1.014)	**3.299 (1.982, 5.490)**	<0.001	**5.099 (2.580, 10.080)**	0.999 (0.984, 1.014)	0.525 (0.290, 0.950)	<0.001
1 Weeks	1.194 (0.603, 2.368)	1.001 (0.992, 1.011)	**3.066 (1.878, 5.005)**	<0.001	**5.519 (2.877, 10.584)**	1.000 (0.986, 1.015)	0.531 (0.301, 0.936)	<0.001
**After pregnancy**								
1 Weeks	1.285 (0.678, 2.432)	1.000 (0.991, 1.009)	**2.731 (1.722, 4.332)**	<0.001	**5.638 (3.073, 10.344)**	1.003 (0.990, 1.016)	0.538 (0.317, 0.912)	<0.001
2 Weeks	1.385 (0.766, 2.506)	1.000 (0.991, 1.008)	**2.353 (1.525, 3.632)**	<0.001	**5.484 (3.120, 9.639)**	1.006 (0.994, 1.017)	0.547 (0.336, 0.892)	<0.001
3 Weeks	1.487 (0.845, 2.617)	1.000 (0.992, 1.009)	**1.979 (1.302, 3.009)**	0.002	**5.122 (2.989, 8.778)**	1.008 (0.998, 1.02)	0.560 (0.352, 0.891)	<0.001
4 Weeks	1.581 (0.897, 2.785)	1.001 (0.993, 1.010)	**1.640 (1.074, 2.502)**	0.021	**4.633 (2.696, 7.963)**	**1.011 (1.000, 1.023)**	0.579 (0.364, 0.921)	<0.001
5 Weeks	1.652 (0.909, 2.999)	1.003 (0.994, 1.011)	1.350 (0.865, 2.106)	0.111	**4.094 (2.312, 7.249)**	**1.014 (1.002, 1.027)**	0.604 (0.370, 0.987)	<0.001

6 Weeks	1.686 (0.886, 3.207)	1.004 (0.995, 1.014)	1.115 (0.692, 1.795)	0.262	**3.563 (1.924, 6.599)**	**1.016 (1.002, 1.031)**	0.639 (0.376, 1.087)	<0.001
7 Weeks	1.670 (0.839, 3.325)	1.006 (0.996, 1.016)	0.932 (0.561, 1.547)	0.337	**3.082 (1.593, 5.961)**	**1.018 (1.003, 1.034)**	0.687 (0.389, 1.214)	0.002
8 Weeks	1.596 (0.779, 3.269)	1.007 (0.997, 1.017)	0.795 (0.470, 1.345)	0.307	**2.670 (1.344, 5.307)**	**1.019 (1.003, 1.036)**	0.751 (0.415, 1.362)	0.014
9 Weeks	1.461 (0.714, 2.992)	1.008 (0.998, 1.018)	0.699 (0.414, 1.182)	0.235	**2.338 (1.178, 4.642)**	**1.020 (1.003, 1.037)**	0.838 (0.463, 1.519)	0.049
10 Weeks	1.284 (0.646, 2.553)	1.008 (0.998, 1.018)	0.635 (0.383, 1.052)	0.157	**2.074 (1.076, 3.999)**	**1.019 (1.004, 1.035)**	0.952 (0.539, 1.682)	0.103
11 Weeks	1.093 (0.576, 2.074)	1.008 (0.999, 1.017)	0.593 (0.370, 0.953)	0.088	**1.859 (1.010, 3.422)**	**1.018 (1.004, 1.032)**	1.095 (0.646, 1.856)	0.149
12 Weeks	0.910 (0.503, 1.647)	1.007 (0.999, 1.016)	0.569 (0.365, 0.885)	0.038	1.678 (0.958, 2.941)	**1.017 (1.004, 1.029)**	1.266 (0.779, 2.057)	0.146
13 Weeks	0.748 (0.426, 1.312)	1.007 (0.999, 1.015)	0.556 (0.364, 0.850)	0.013	1.522 (0.899, 2.575)	**1.015 (1.004, 1.026)**	1.464 (0.928, 2.309)	0.093
14 Weeks	0.612 (0.350, 1.073)	1.006 (0.998, 1.014)	0.554 (0.363, 0.845)	0.005	1.381 (0.822, 2.318)	**1.013 (1.002, 1.023)**	**1.682 (1.071, 2.640)**	0.044
15 Weeks	0.504 (0.280, 0.909)	1.005 (0.997, 1.013)	0.558 (0.359, 0.869)	0.002	1.251 (0.729, 2.145)	1.011 (0.999, 1.022)	**1.908 (1.190, 3.060)**	0.023
16 Weeks	0.422 (0.224, 0.796)	1.004 (0.995, 1.012)	0.568 (0.354, 0.912)	0.002	1.127 (0.631, 2.011)	1.009 (0.996, 1.022)	**2.125 (1.276, 3.539)**	0.016
17 Weeks	0.362 (0.183, 0.716)	1.003 (0.993, 1.012)	0.582 (0.351, 0.963)	0.001	1.007 (0.541, 1.876)	1.007 (0.993, 1.022)	**2.308 (1.333, 3.998)**	0.013
18 Weeks	0.321 (0.157, 0.655)	1.002 (0.992, 1.011)	0.596 (0.352, 1.007)	0.001	0.890 (0.465, 1.706)	1.006 (0.991, 1.022)	**2.432 (1.367, 4.324)**	0.010
19 Weeks	0.297 (0.145, 0.609)	1.001 (0.991, 1.011)	0.609 (0.360, 1.032)	0.001	0.776 (0.404, 1.491)	1.006 (0.991, 1.021)	**2.47 (1.384, 4.406)**	0.007
20 Weeks	0.287 (0.144, 0.574)	1.000 (0.991, 1.010)	0.621 (0.373, 1.034)	<0.001	0.667 (0.355, 1.253)	1.007 (0.992, 1.021)	**2.421 (1.385, 4.233)**	0.004
21 Weeks	0.288 (0.150, 0.552)	1.000 (0.991, 1.009)	0.631 (0.390, 1.021)	<0.001	0.567 (0.315, 1.021)	1.007 (0.994, 1.021)	**2.299 (1.363, 3.880)**	0.001
22 Weeks	0.299 (0.163, 0.549)	1.000 (0.992, 1.008)	0.640 (0.407, 1.006)	<0.001	0.476 (0.276, 0.821)	1.009 (0.997, 1.021)	**2.124 (1.307, 3.454)**	<0.001
23 Weeks	0.319 (0.177, 0.574)	1.000 (0.992, 1.008)	0.647 (0.417, 1.004)	<0.001	0.396 (0.235, 0.667)	1.011 (0.999, 1.023)	**1.916 (1.201, 3.059)**	<0.001
24 Weeks	0.348 (0.188, 0.646)	1.000 (0.991, 1.009)	0.654 (0.413, 1.034)	0.001	0.327 (0.190, 0.563)	**1.013 (1.000, 1.027)**	**1.695 (1.038, 2.768)**	<0.001
25 Weeks	0.388 (0.191, 0.788)	1.000 (0.990, 1.010)	0.659 (0.392, 1.109)	0.009	0.269 (0.144, 0.501)	1.016 (0.999, 1.032)	1.476 (0.839, 2.596)	<0.001
26 Weeks	0.438 (0.186, 1.028)	1.000 (0.989, 1.012)	0.664 (0.358, 1.233)	0.071	0.220 (0.103, 0.467)	1.019 (0.998, 1.040)	1.270 (0.640, 2.518)	<0.001
27 Weeks	0.499 (0.177, 1.407)	1.001 (0.987, 1.015)	0.669 (0.317, 1.408)	0.240	0.179 (0.071, 0.449)	1.022 (0.996, 1.048)	1.084 (0.470, 2.500)	0.001
28 Weeks	0.571 (0.165, 1.980)	1.001 (0.985, 1.017)	0.673 (0.277, 1.636)	0.461	0.146 (0.048, 0.442)	1.025 (0.994, 1.057)	0.921 (0.337, 2.520)	0.003

Abbreviations: GDM, gestational diabetes mellitus; GDMA1, diet-controlled GDM; GDMA2, insulin-treated GDM; OR, odds ratio; 95% CI, 95% confidence interval

Note: Weekly average temperature was divided into three levels using the 5th and 95th percentiles.

Distributed lag non-linear models (DLNMs) incorporated with logistic regression was used to calculate adjusted ORs (95% CIs) each 10μg/m^3 increment in the concentrations of O_3 at a weekly level over the preconception period and pregnancy.
All models adjusted for maternal age, preconception BMI, educational level, occupation, gravidity, parity, conception season, gestational hypertension, newborn gender, and natural cubic splines with 3 degrees of freedom for dew point.
Black bold indicate statistically significance ($P < 0.05$) with positive effects.

Table S11. Sensitivity analysis of effects of O_3 exposure on GDMA1 and GDMA2 between the pre-pandemic period (2017–2019) and the pandemic period (2020–2023)

Exposure	Before COVID-19 pandemic (2017-2019)		COVID-19 pandemic (2017-2019)	
	GDMA1	GDMA2	GDMA1	GDMA2
	Adjusted OR (95% CI)	Adjusted OR (95% CI)	Adjusted OR (95% CI)	Adjusted OR (95% CI)
**Preconception**				
12 Weeks	**1.055 (1.005, 1.106)**	1.128 (0.973, 1.308)	1.017 (0.954, 1.085)	1.033 (0.824, 1.293)
11 Weeks	1.041 (0.999, 1.085)	**1.145 (1.01, 1.298)**	1.011 (0.956, 1.069)	1.027 (0.848, 1.243)
10 Weeks	1.029 (0.993, 1.066)	**1.162 (1.045, 1.292)**	1.005 (0.958, 1.054)	1.022 (0.869, 1.201)
9 Weeks	1.017 (0.985, 1.049)	**1.179 (1.076, 1.291)**	0.999 (0.958, 1.041)	1.017 (0.885, 1.169)
8 Weeks	1.006 (0.977, 1.035)	**1.195 (1.101, 1.296)**	0.993 (0.957, 1.031)	1.013 (0.893, 1.148)
7 Weeks	0.995 (0.969, 1.023)	**1.21 (1.118, 1.309)**	0.988 (0.954, 1.024)	1.009 (0.893, 1.14)
6 Weeks	0.987 (0.961, 1.014)	**1.224 (1.129, 1.326)**	0.984 (0.95, 1.019)	1.007 (0.888, 1.141)
5 Weeks	0.98 (0.953, 1.007)	**1.236 (1.136, 1.345)**	0.981 (0.946, 1.017)	1.006 (0.881, 1.148)
4 Weeks	0.974 (0.947, 1.003)	**1.247 (1.141, 1.362)**	0.978 (0.942, 1.016)	1.006 (0.876, 1.156)
3 Weeks	0.971 (0.943, 1)	**1.255 (1.146, 1.374)**	0.977 (0.94, 1.016)	1.008 (0.875, 1.162)
2 Weeks	0.97 (0.942, 0.998)	**1.26 (1.151, 1.38)**	0.978 (0.94, 1.016)	1.012 (0.879, 1.165)
1 Weeks	0.971 (0.944, 0.999)	**1.264 (1.158, 1.379)**	0.979 (0.943, 1.017)	1.018 (0.889, 1.166)
**After pregnancy**				
1 Weeks	0.974 (0.947, 1.001)	**1.265 (1.164, 1.374)**	0.982 (0.946, 1.019)	1.026 (0.903, 1.166)
2 Weeks	0.978 (0.953, 1.005)	**1.265 (1.17, 1.368)**	0.986 (0.951, 1.021)	1.036 (0.918, 1.169)
3 Weeks	0.984 (0.959, 1.01)	**1.265 (1.172, 1.364)**	0.99 (0.956, 1.025)	1.047 (0.932, 1.176)
4 Weeks	0.99 (0.965, 1.016)	**1.265 (1.172, 1.365)**	0.994 (0.96, 1.03)	1.06 (0.943, 1.192)
5 Weeks	0.997 (0.97, 1.024)	**1.266 (1.169, 1.371)**	0.999 (0.964, 1.036)	1.074 (0.951, 1.214)
6 Weeks	1.003 (0.976, 1.031)	**1.27 (1.167, 1.382)**	1.004 (0.967, 1.042)	1.09 (0.957, 1.242)
7 Weeks	1.009 (0.981, 1.039)	**1.276 (1.167, 1.395)**	1.008 (0.97, 1.048)	1.107 (0.964, 1.272)

8 Weeks	1.014 (0.985, 1.045)	**1.286 (1.172, 1.411)**	1.012 (0.972, 1.052)	1.126 (0.975, 1.299)
9 Weeks	1.018 (0.988, 1.049)	**1.3 (1.185, 1.426)**	1.014 (0.975, 1.055)	1.145 (0.992, 1.322)
10 Weeks	1.02 (0.991, 1.05)	**1.319 (1.206, 1.442)**	1.016 (0.977, 1.056)	**1.165 (1.014, 1.339)**
11 Weeks	1.021 (0.993, 1.05)	**1.341 (1.232, 1.459)**	1.017 (0.979, 1.056)	**1.185 (1.039, 1.353)**
12 Weeks	1.021 (0.994, 1.049)	**1.365 (1.26, 1.478)**	1.017 (0.981, 1.054)	**1.205 (1.064, 1.366)**
13 Weeks	1.021 (0.994, 1.047)	**1.39 (1.288, 1.499)**	1.017 (0.982, 1.053)	**1.225 (1.086, 1.38)**
14 Weeks	1.019 (0.993, 1.046)	**1.414 (1.311, 1.524)**	1.016 (0.981, 1.052)	**1.242 (1.103, 1.399)**
15 Weeks	1.018 (0.992, 1.045)	**1.436 (1.329, 1.552)**	1.016 (0.98, 1.052)	**1.258 (1.112, 1.422)**
16 Weeks	1.016 (0.989, 1.044)	**1.455 (1.341, 1.579)**	1.015 (0.979, 1.053)	**1.27 (1.116, 1.446)**
17 Weeks	1.015 (0.987, 1.044)	**1.469 (1.348, 1.602)**	1.015 (0.978, 1.054)	**1.28 (1.116, 1.468)**
18 Weeks	1.014 (0.985, 1.044)	**1.477 (1.35, 1.616)**	1.016 (0.977, 1.055)	**1.285 (1.114, 1.482)**
19 Weeks	1.014 (0.985, 1.044)	**1.476 (1.348, 1.616)**	1.017 (0.978, 1.056)	**1.286 (1.113, 1.485)**
20 Weeks	1.014 (0.986, 1.044)	**1.468 (1.344, 1.603)**	1.018 (0.981, 1.057)	**1.282 (1.113, 1.476)**
21 Weeks	1.016 (0.988, 1.044)	**1.452 (1.335, 1.58)**	1.02 (0.984, 1.058)	**1.274 (1.113, 1.458)**
22 Weeks	1.017 (0.991, 1.045)	**1.431 (1.322, 1.549)**	1.023 (0.988, 1.06)	**1.263 (1.111, 1.435)**
23 Weeks	1.02 (0.994, 1.046)	**1.406 (1.303, 1.516)**	1.026 (0.992, 1.062)	**1.249 (1.105, 1.411)**
24 Weeks	1.022 (0.996, 1.049)	**1.376 (1.275, 1.486)**	1.03 (0.996, 1.066)	**1.232 (1.09, 1.393)**
25 Weeks	1.025 (0.997, 1.053)	**1.344 (1.237, 1.461)**	1.034 (0.998, 1.071)	**1.214 (1.065, 1.384)**
26 Weeks	1.028 (0.998, 1.06)	**1.311 (1.191, 1.442)**	1.038 (0.999, 1.079)	**1.195 (1.03, 1.387)**
27 Weeks	1.032 (0.997, 1.067)	**1.276 (1.14, 1.429)**	1.042 (0.998, 1.089)	1.176 (0.989, 1.398)
28 Weeks	1.035 (0.995, 1.076)	**1.242 (1.087, 1.419)**	1.047 (0.996, 1.1)	1.156 (0.943, 1.416)

Abbreviations: GDM, gestational diabetes mellitus; GDMA1, diet-controlled GDM; GDMA2, insulin-treated GDM; OR, odds ratio; 95% CI, 95% confidence interval

Note: Distributed lag non-linear models (DLNMs) incorporated with logistic regression was used to calculate adjusted ORs (95% CIs) each 10μg/m^3 increment in the concentrations of O_3 at a weekly level over the preconception period and pregnancy.

All models adjusted for maternal age, preconception BMI, educational level, occupation, gravidity, parity, conception season, gestational hypertension, newborn gender, $PM_{2.5}$ concentration and natural cubic splines with 3 degrees of freedom for ambient temperature and dew point. Black bold indicate statistically significance ($P < 0.05$) with positive effects.

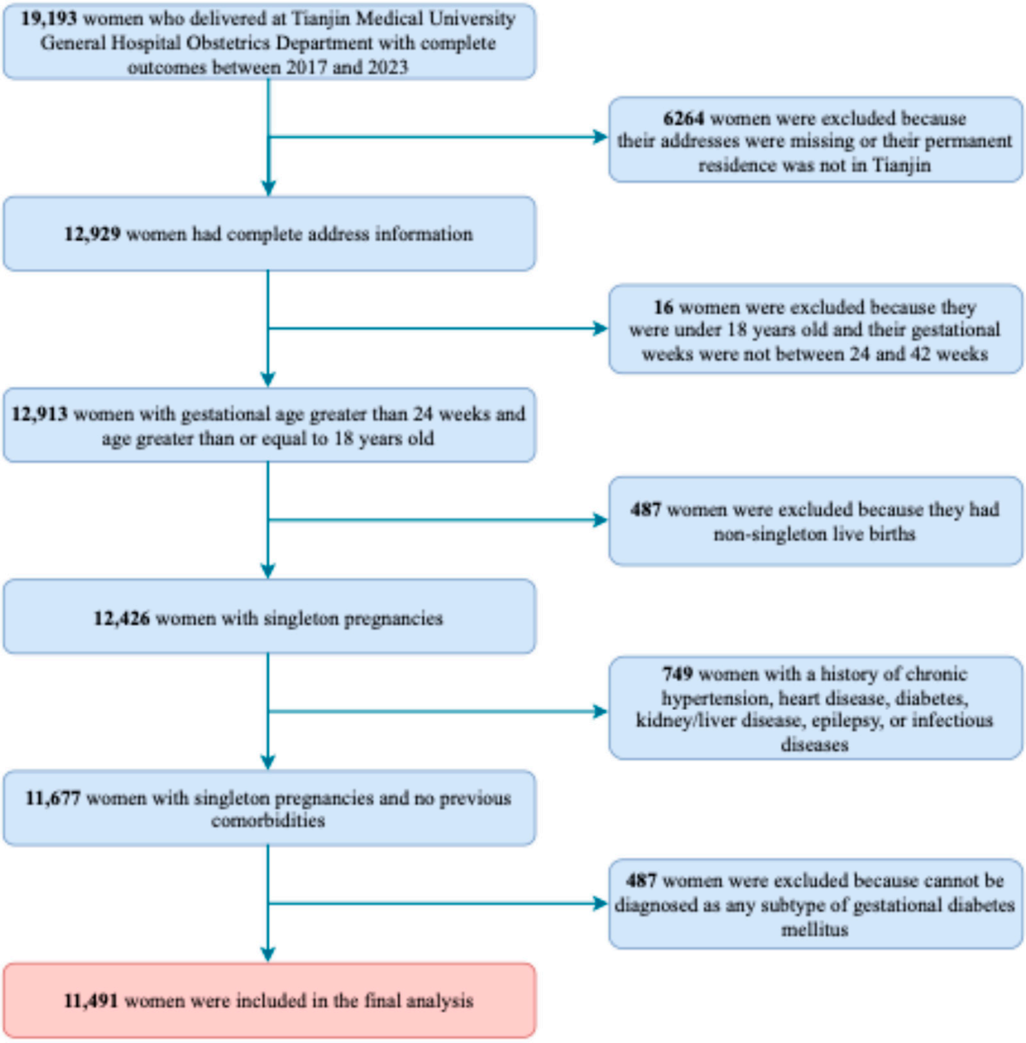

Figure S1. The inclusion and exclusion process of the study population
